# Supplementary material for: Ablative radiotherapy improves survival but does not cure autochthonous mouse models of prostate and colorectal cancer
Source: Commun Med (Lond). 2023 Aug 9;3:108. doi: 10.1038/s43856-023-00336-3 (PMC10412558; doi:10.1038/s43856-023-00336-3)

Supplementary Fig. 1: Nanodot dosimeter and radiochromic film exposure setup.

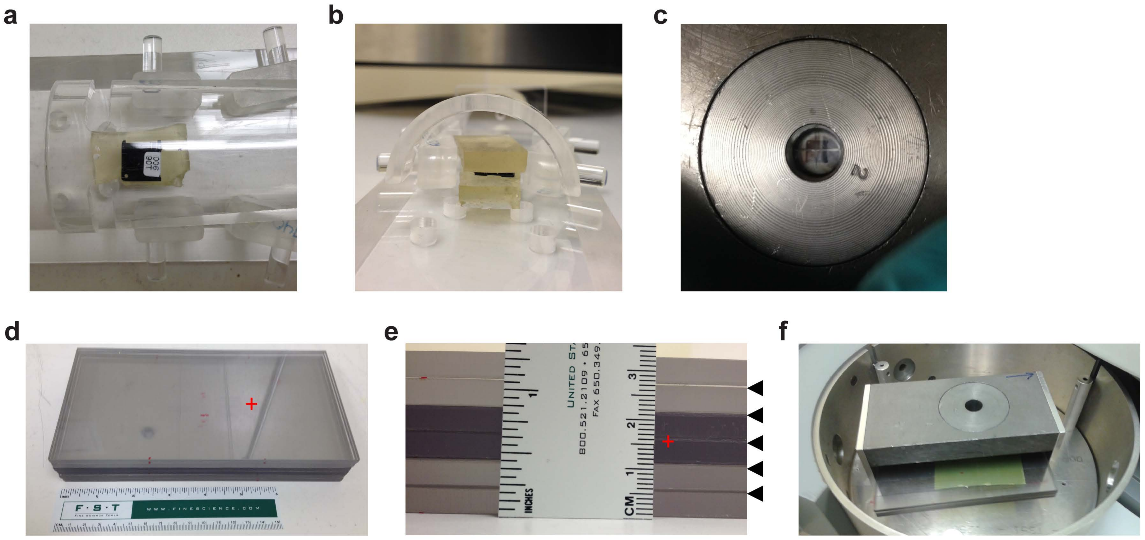

a Top view of Nanodot dosimeter exposure setup. The upper bolus slab is omitted for visualization of the dosimeter. **b** Side view of dosimeter exposure setup. **c** Top view of dosimeter in collimated field. The upper bolus slab is omitted for visualization of the dosimeter. **d** Top view of radiochromic film exposure setup. The red cross indicates the location at which film was centered to measure the horizontal dose distribution. **e** Side view of radiochromic film exposure setup. The red cross indicates the location at which film was centered to measure the horizontal dose distribution. The black arrowheads indicate the level at which film was placed to determine the dose distribution in the perpendicular plane. **f** Placement of radiochromic film in the collimated field with upper stack of plexiglass sheets removed for visualization of film.

Supplementary Fig. 2: Flow cytometry gating strategy.

a

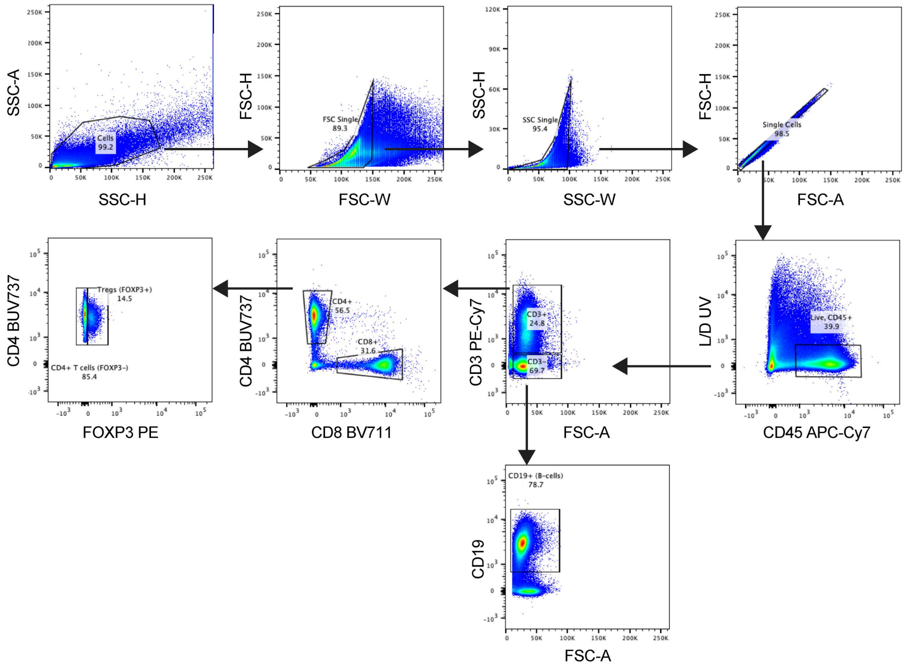

a Representative gating strategy used in flow cytometry analysis of lymphocytes in blood, spleen, and lymph nodes (spleen sample shown). See methods for details.

Supplementary Fig. 3: Design and validation of a mouse restrainer for stereotactic ablative radiotherapy (SART) delivery.

a

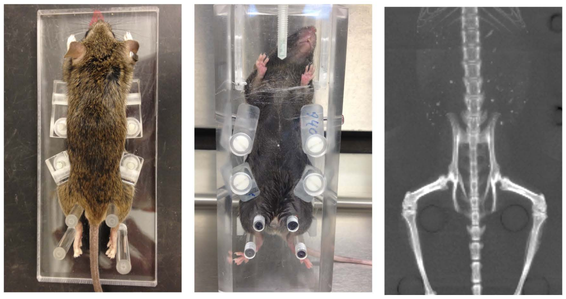

b

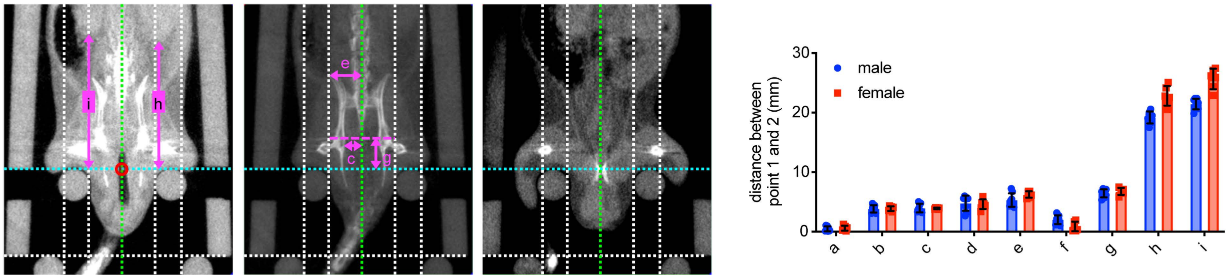

	Point 1	Point 2	F 24-25g	F 26-28g	F 29-32g	F 33-34g	F 35-40g	F 41-47g	M 24-25g	M 26-28g	M 29-32g	M 33-34g	M 35-40g	M 41-47g
a	Pubic symphysis	Sagittal ref. plane	0.0, 0.9	0.4, 1.2	0.3, 1.2	0.4, 0.6	0.3, 0.6	0.1, 0.4	0.0, 0.7	0.1, 1.1	0.2, 1.0	0.2, 0.3	0.1, 0.5	0.2, 0.3
b	Medial edge of left fem. head	Sagittal ref. plane	3.4, 3.8	3.6, 4.3	3.3, 4.2	3.1, 4.1	3.5, 4.8	3.8, 4.1	3.2, 3.8	3.1, 4.4	3.8, 4.7	4.0, 4.5	3.6, 4.2	4.3, 4.4
c	Medial edge of right fem. head	Sagittal ref. plane	3.7, 4.1	3.8, 4.0	3.8, 4.0	4.0, 5.0	3.9, 4.7	4.3, 4.9	2.8, 3.9	3.2, 5.0	3.3, 3.9	3.6, 3.8	4.2, 4.5	4.7, 4.8
d	Lateral edge of left iliac crest	Sagittal ref. plane	3.4, 3.7	3.4, 4.8	4.4, 5.9	4.4, 4.6	5.1, 6.0	4.7, 5.0	4.5, 6.5	2.7, 5.6	5.5, 6.0	6.3, 6.4	5.1, 5.6	5.1, 5.7
e	Lateral edge of right iliac crest	Sagittal ref. plane	6.2, 7.1	6.1, 7.0	5.5, 6.5	5.8, 6.6	5.1, 5.4	6.0, 6.2	3.7, 5.2	4.1, 7.2	4.7, 5.2	4.1, 4.2	5.1, 6.0	6.1, 6.0
f	Top of pubic symphysis	Axial ref. plane	0.4, 2.6	0.4, 1.9	0.1, 1.1	0.4, 1.2	0.1, 1.6	1.1, 1.9	0.1, 0.4	1.0, 3.1	2.0, 2.6	2.1, 2.9	2.4, 3.6	2.1, 4.1
g	Upper edge of fem. heads	Axial ref. plane	4.8, 6.0	6.4, 7.1	5.8, 7.5	6.7, 7.3	6.2, 7.3	8.1, 8.4	4.8, 5.7	5.3, 7.1	6.3, 7.2	6.7, 7.1	6.7, 8.0	7.7, 9.5
h	Inferior pole of left kidney	Axial ref. plane	19.7, 21.5	20.5, 25.1	21.8, 24.2	22.5, 27.1	23.3, 24.3	24.4, 25.3	18.7, 20.2	17.5, 20.6	19.0, 20.1	21.1, 23.4	22.7, 24.1	20.6, 22.1
i	Inferior pole of right kidney	Axial ref. plane	22.4, 23.9	22.9, 27.1	25.9, 27.1	25.7, 28.0	25.4, 27.9	27.2, 28.9	19.6, 21.9	19.9, 22.4	20.7, 22.4	23.0, 25.1	23.3, 25.6	24.3, 25.1

a Initial prototype of restrainer and mean intensity projection (MIP) CT image of the lower torso and hind limbs showing optimal positioning of the pelvis and immobilization of hind limbs. **b** Quantitative assessment of reproducible animal positioning relative to reference points on the restrainer. Representative images show the anatomic and reference land-marks evaluated. Letters correspond to measured distances between points described in the table. The sagittal reference plane (shown in green) is defined as a straight-line midpoint between the medial edge of the upper and lower pins. The axial reference plane (shown in cyan) is defined as a straight line connecting the upper edge of the upper pins. The central axis of the field (red circle in left panel) is defined as the intersection of the sagittal and axial reference planes. Values show in the table are the minimum and maximum distances (in mm) observed between point 1 and 2. The top row indicates the sex (F, female; M, male) and body weight range (g, grams). To know whether results would be generalizable to mice of different strains, we used mixed-strain (C57BL/6J; 129/SvJ), inbred (C57BL/6J), and outbred (CD-1) strains for this analysis. All mice were age 3 months or older. Shown in the graph are distances (mean +/- SD) between point 1 and 2 as described in the table for mice weighing 26 to 32 grams (n=8 for male, n=6 for female).

Supplementary Fig. 4: Localizing the prostate relative to restrainer landmarks.

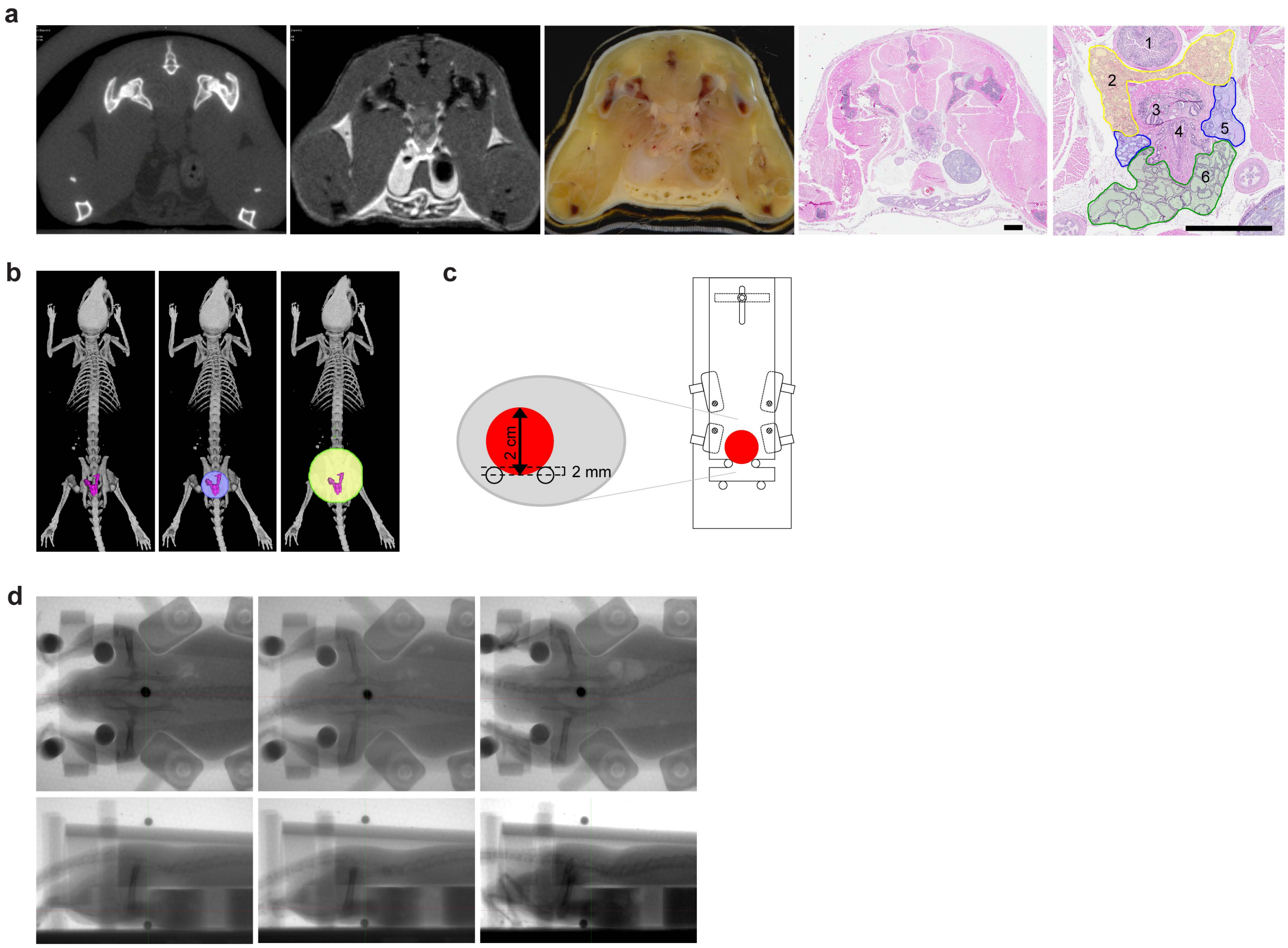

a In situ anatomy of the prostate shown on formalin-fixed paraffin-embedded tissue sections of a mouse previously evaluated in the same anatomic position by CT, MRI and wet mount cross-sectional imaging. 1, rectum; 2, dorsal prostate; 3, ducts from anterior prostate and seminal vesicles (both of which are located cranial to the axial slice shown); 4, urethra; 5, lateral prostate; 6, ventral prostate. Scale bar = 2 mm. **b** Location of the prostate relative to bony anatomic landmarks (see text for details). Relationship of the prostate to 1 and 2 cm diameter pelvic radiation fields is shown. **c** Optimal alignment of the restrainer with the collimator aperture in order to position the prostate within a 2 cm radiation field. **d** Fluoroscopic images (top and side view) of three male mice immobilized in the restrainer. The radio-opaque markers on the restrainer were used to indicate the center of the radiation field. Note that the bony pelvis is accurately aligned to place the geometric center of the prostate at the center of the beam.

Supplementary Fig. 5: Dosimetry and modeling of radiation dose distribution in mice.

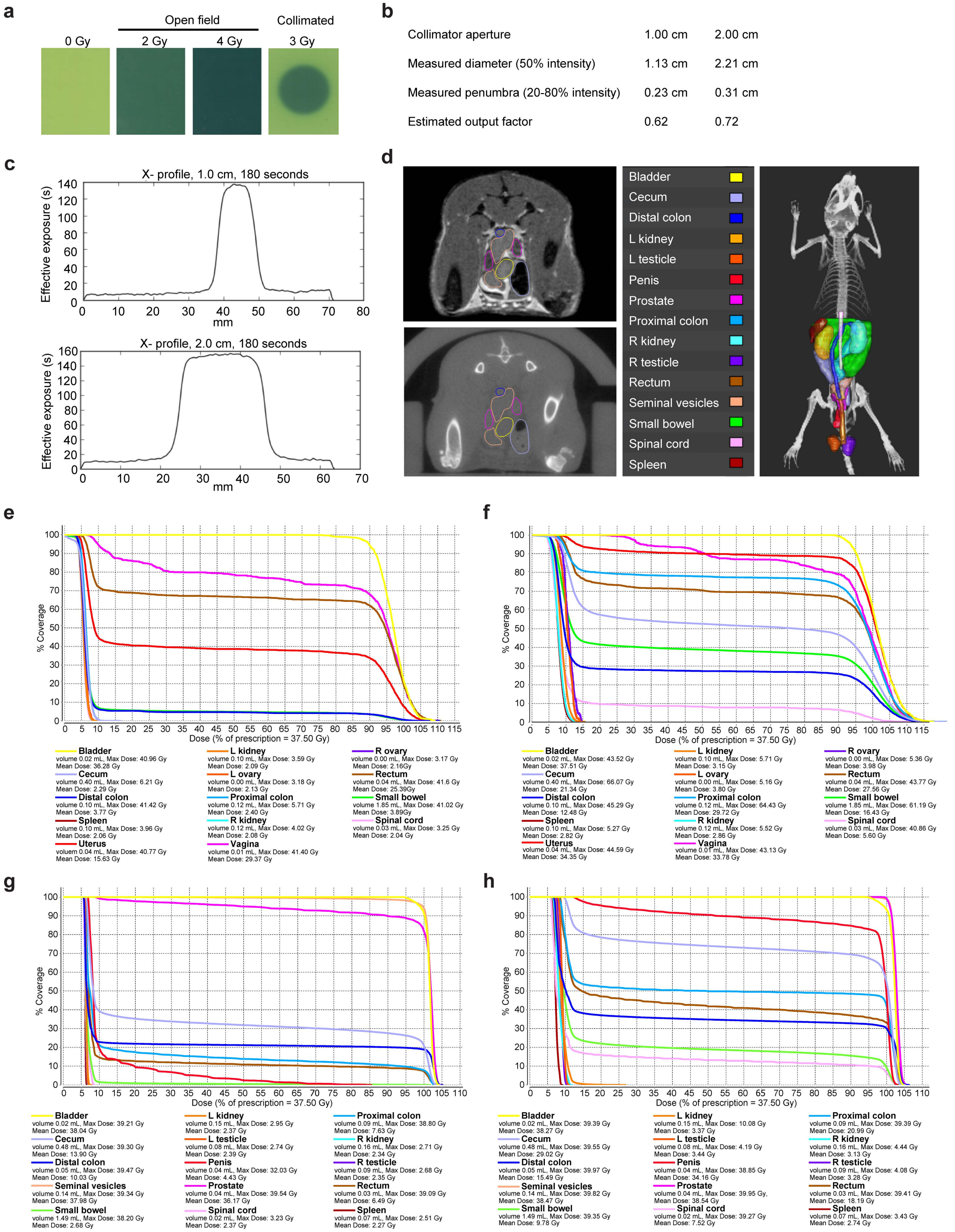

a Representative result of radiochromic film exposed in an open field at 2 dose levels and a 2 cm collimated field.
b Collimated field parameters evaluated by radiochromic film dosimetry. The average of two independent tests is shown.
c Representative plots of the horizontal dose distribution for circular collimated fields with 1 and 2 cm apertures evaluated at mid-plane of the radiation field by radiochromic film dosimetry. **d** Representative MRI and µCT images demonstrating how rigid image registration was used to transfer abdominopelvic organs contoured on MRI (upper left) to µCT (lower left).

The panel at right shows a dorsal view 3D rendering of abdominopelvic organs of a male mouse defined by MRI onto the bony skeleton imaged by μCT. **e** Dose-volume histogram for a 1 cm pelvic radiation field in a female mouse. **f** Dose-volume histogram for a 2 cm pelvic radiation field in a female mouse. **g** Dose-volume histogram for a 1 cm pelvic radiation field in a male mouse. **h** Dose-volume histogram for a 2 cm pelvic radiation field in a male mouse.

Supplementary Fig. 6: Evaluating dose limiting toxicity of hypofractionated stereotactic ablative radiotherapy (SART) to the pelvis.

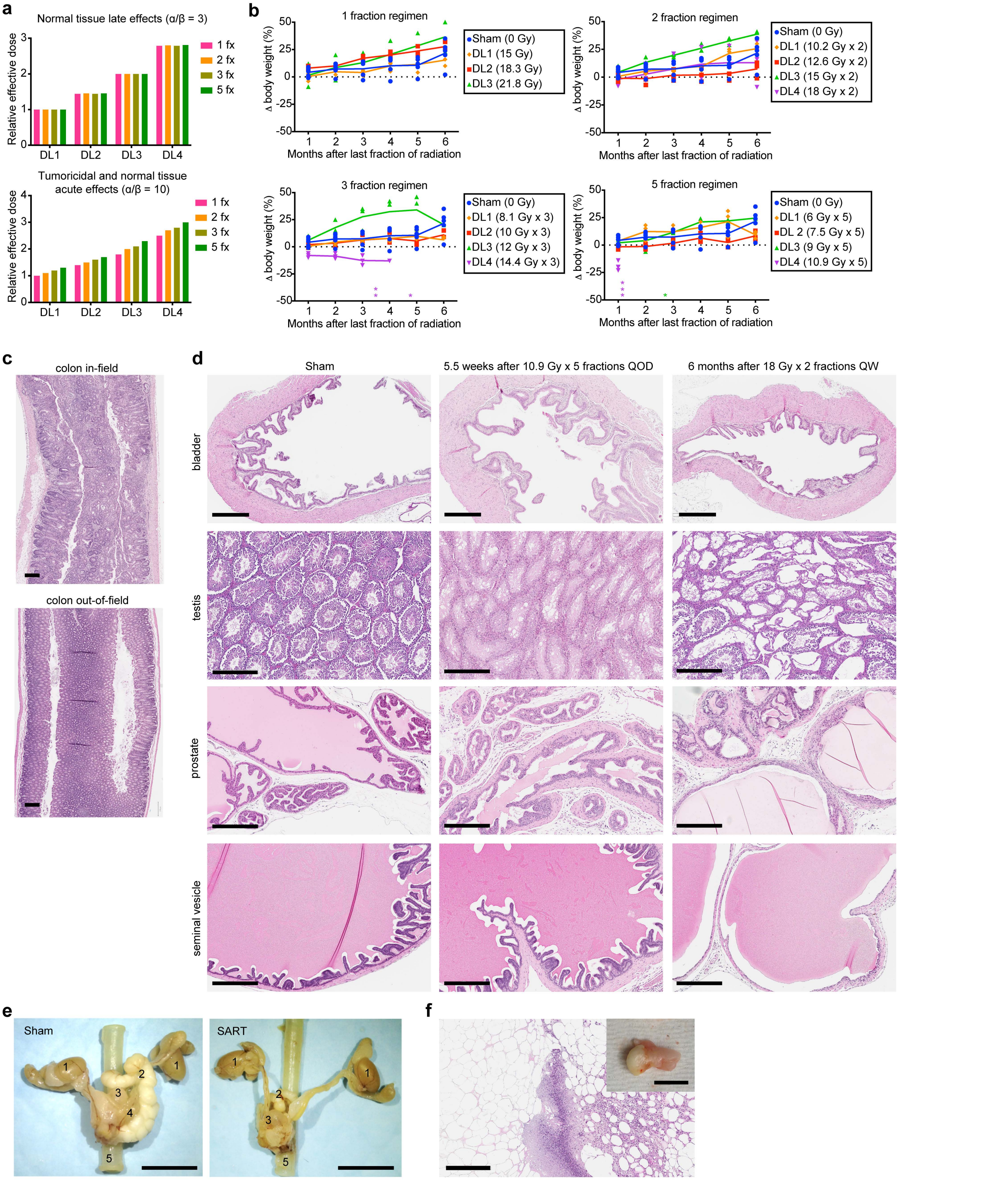

a Graphical representation of the relative biologic effective dose (BED) for the dose levels shown in table 1. BED was calculated using alpha-beta ratio = 3 for late effects and alpha-beta ratio = 10 for acute effects and plotted relative to the BED for a single fraction at DL1. **b** Body weight change relative to pretreatment baseline. Dose level (DL) and RT schedule are defined in Table 1. Individual animals are shown with group means connected. All subjects are male. N=5 for 0 Gy,

n=3 for all others (except n=2 for 21.8 Gy single fraction group, as one subject was removed from study due to injury unrelated to treatment). Each asterisk (*) indicates time of death/euthanasia of a single subject. **c** Representative histology of distal colon inside the radiation field (in-field) and adjacent colon outside the radiation field (out-of-field) 5 weeks after treatment with 5 fractions of 10.9 Gy. **d** Representative histology of GU tissue 5.5 weeks after 10.9 Gy x 5 fractions QOD (every other day) or 6 months after 18 Gy x 2 fractions QW (once a week) to a 2 cm pelvic radiation field. GU tissue from a sham treated control is shown for comparison. Scale bar = 600 μm for bladder and 300 μm for all others. **e** Gross pathology of genitourinary organs and adjacent rectum of male mouse 6 months after treatment with 18 Gy x 2 fractions QW to a 2 cm pelvic radiation field centered on the prostate. Sham treated control is shown for comparison. 1: testis, 2: seminal vesicles, 3: bladder, 4: anterior prostate, 5: rectum. Note: anterior prostate is small and obscured by the bladder in the SART treated specimen. Scale bar 3 mm. **f** Histology of epididymal fat pad showing region of fibrosis and necrosis adjacent to normal fatty tissue at 6 months after treatment with 18 Gy x 2 fractions QW to a 2 cm pelvic radiation field. Scale bar = 300 μm. Gross pathology is shown in the inset (scale bar = 5 mm).

Supplementary Fig. 7: Acute and chronic effects of hypofractionated stereotactic ablative radiotherapy (SART) to the pelvis and lower abdomen.

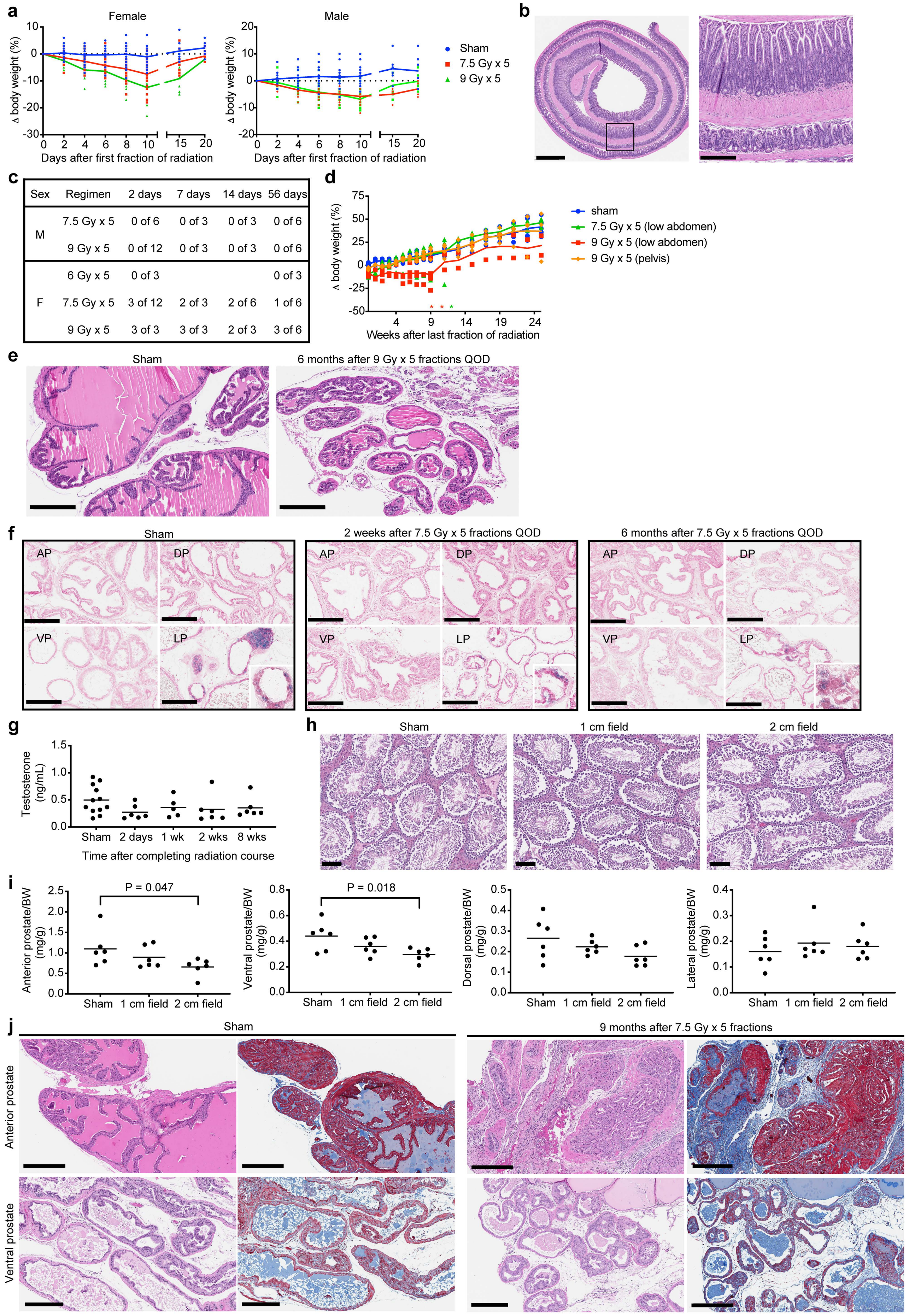

Sex	Regimen	2 days	7 days	14 days	56 days
M	7.5 Gy x 5	0 of 6	0 of 3	0 of 3	0 of 6
	9 Gy x 5	0 of 12	0 of 3	0 of 3	0 of 6
F	6 Gy x 5	0 of 3			0 of 3
	7.5 Gy x 5	3 of 12	2 of 3	2 of 6	1 of 6
	9 Gy x 5	3 of 3	3 of 3	2 of 3	3 of 6

a Body weight change relative to pretreatment baseline during the first 3 weeks of treatment with 7.5 Gy or 9 Gy x 5 fractions QOD. Mean +/- SEM. Females n=12 for days 2–10, n=6 for days 15 and 20. Males n=15 for days 2–10, n=6 for days 15 and 20. **b** Swiss roll of ilium with distal end at center showing hyperplasia of the mucosa and muscularis propria 11 weeks after completing 9 Gy x 5 fractions QOD to a 2 cm pelvic radiation field. Corresponds to the gross specimen shown in Fig. 3f. Scale bar = 1 mm for the low power view and 300 µm for the high-power view. **c** Incidence of acute small bowel toxicity assessed at the indicated time points after completing radiation to a 2 cm pelvic radiation field (7.5 Gy or 9 Gy x 5 fractions QOD). Toxicity was defined as any abnormal appearance of the intestine or luminal contents determined by visual inspection at time of necropsy. **d** Body weight change relative to pretreatment baseline for female mice treated with a 1 cm radiation field targeted to the pelvis (9 Gy x 5 fractions QOD) or low abdomen (7.5 or 9 Gy x 5 fractions QOD). Individual animals are shown with group means connected. n=5 for sham group, n=4 for all treatment groups. Each asterisk (*) indicates time of death or protocol mandated euthanasia of a single subject. **e** Representative histology of anterior prostate and seminal vesicle 6 months after 9 Gy x 5 fractions QOD to a 2 cm pelvic radiation field. Sham treated control is shown for comparison. Scale bar = 300 µm. **f** Representative SA-βgal staining of prostate tissue. AP, anterior prostate; DP, dorsal prostate; VP, ventral prostate; LP, lateral prostate. Scale bar = 200 µm. Irrespective of treatment group, some LP glands had βgal positive cells as shown at high power magnification in the inset. **g** Serum testosterone in C57BL/6J male mice at the indicated time points after treatment with 7.5 or 9 Gy x 5 fractions QOD to a 2 cm pelvic radiation field centered on the prostate. Sham treated control is shown for comparison. Individual animals are shown with group means, n=5-12 per group. **h** Representative histology of testis from CD-1 mice 9 months after sham radiation or 7.5 Gy x 5 fractions QOD to a 1 or 2 cm radiation field centered over the prostate. Scale bar = 100 µm. **i** Wet weight of prostate relative to body weight (BW) in male mice 9 months after sham treatment or 7.5 Gy x 5 fractions QOD to a 1 or 2 cm pelvic radiation field. Individual animals are shown with group means, n=6 per group. P value estimate by unpaired, two-tailed t test. **j** Representative H&E and trichrome stain showing fibrosis (blue) between glands in the anterior and ventral prostate of CD-1 male mice 9 months after sham treatment or 7.5 Gy x 5 fractions QOD to a 2 cm pelvic field. Note that secretions in the prostate lumen also stain blue. Scale bar = 300 µm.

Supplementary Fig. 8: Pelvic hypofractionated stereotactic ablative radiotherapy (SART) effects on spleen and lymph nodes.

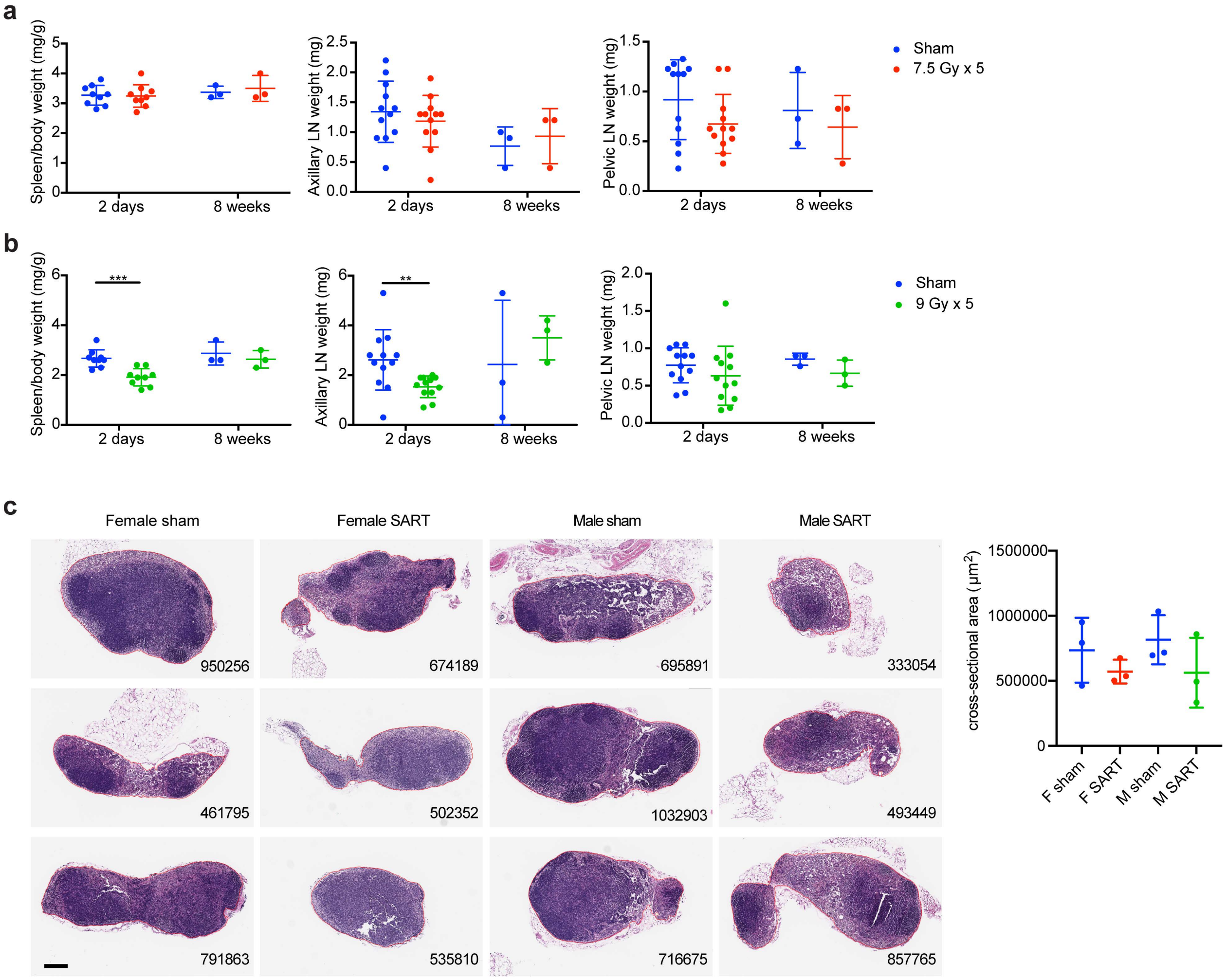

a Weight of whole spleen, axillary lymph node, and pelvic lymph node tissue from female mice. Wet weight was determined at time of necropsy. n=9 for spleen and n=12 for lymph nodes at 2-day time point, n=3 for spleen and lymph nodes at 8-week time point. Mean +/- SD is shown. **b** Weight of whole spleen, axillary lymph node, and pelvic lymph node tissue from male mice. Wet weight was determined at time of necropsy. n=9 for spleen and n=12 for lymph nodes at 2-day time point, n=3 for spleen and lymph nodes at 8-week time point. Mean +/- SD is shown. $^{**}p<0.01$, $^{***}p<0.001$ by unpaired 2-tailed t test. **c** Cross-section at midplane of pelvic lymph nodes (n=3) 2 days after sham treatment or 5 fractions of 7.5 Gy (female) or 9 Gy (male). Number at bottom right indicates the area (µm2) within the red outline, which is summarized in the graph on the right as mean +/- SD with individual data points shown. All images are the same magnification. Scale bar = 200 µm.

Supplementary Fig. 9: Clinical and pathological response of flank xenografts to stereotactic ablative radiotherapy (SART).

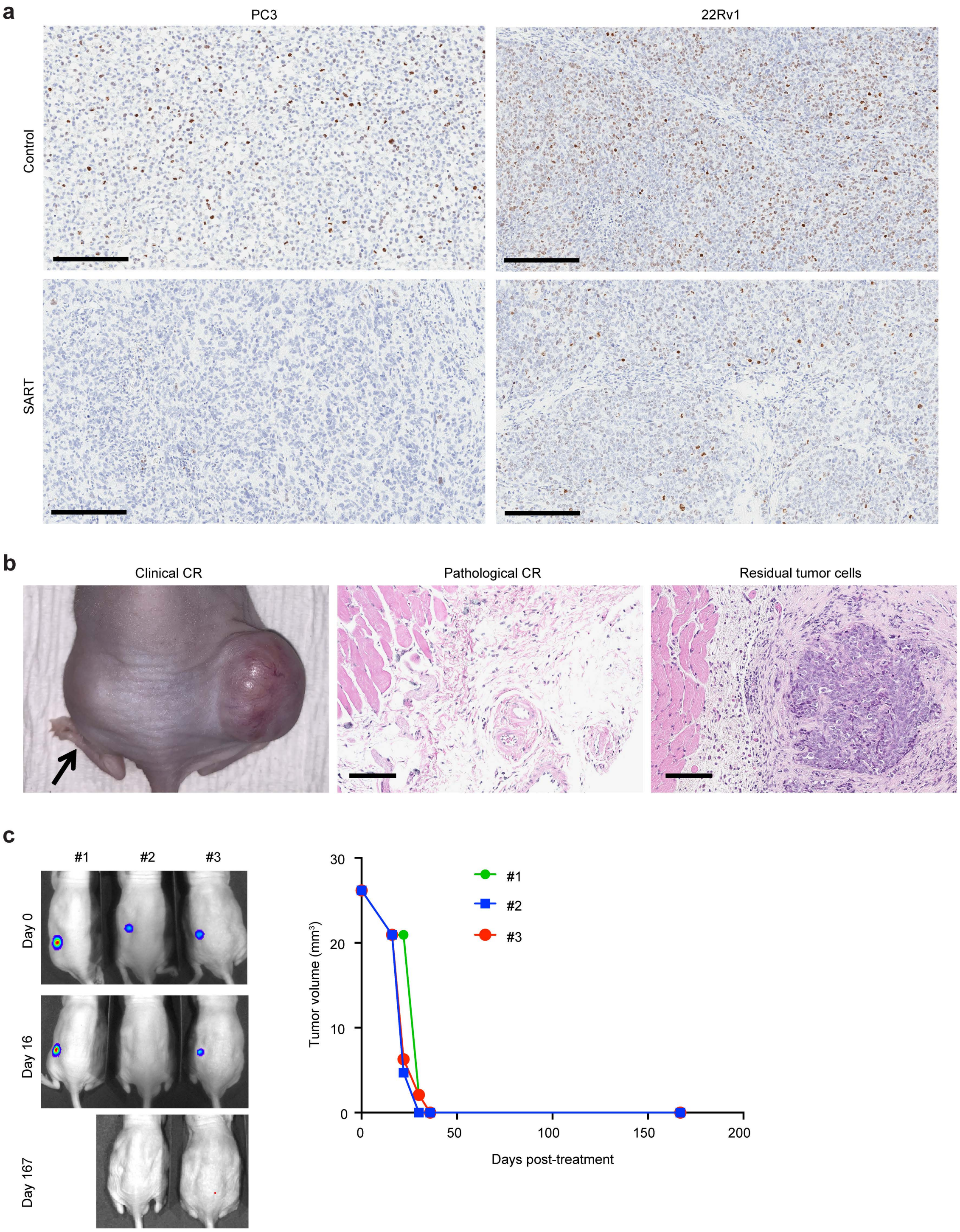

a Representative Ki67 IHC of PC3 and 22Rv1 xenografts one month after 4 fractions of 6 Gy. Untreated control is shown for comparison. Scale bar = 200 µm. **b** Representative examples of clinical and pathological complete responses 4 weeks after completing SART. Clinical complete response was defined as no visible or palpable tumor in the irradiated flank (black arrow). Pathological complete response was defined as no identifiable tumor cells on two step sections through the tumor scar. A SART-treated 22Rv1 xenograft with residual tumor cells is shown for comparison. Scale bar = 100 µm.
c Three nude mice bearing a single PC3-luc xenograft measuring at least 25 mm3 were treated with 5 fractions of 7.5 Gy. Bioluminescence images obtained prior to fraction 1 and on day 16 and 167 thereafter. One animal was moribund on day 138. Necropsy showed no tumors and no evidence of radiation toxicity. Graph shows tumor volume based on caliper measurements.

Supplementary Fig. 10: Radiographic and histopathologic response of prostate cancer to stereotactic ablative radiotherapy (SART).

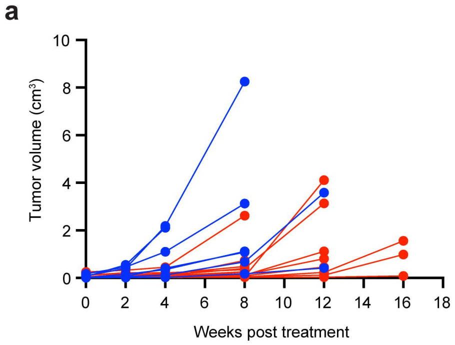

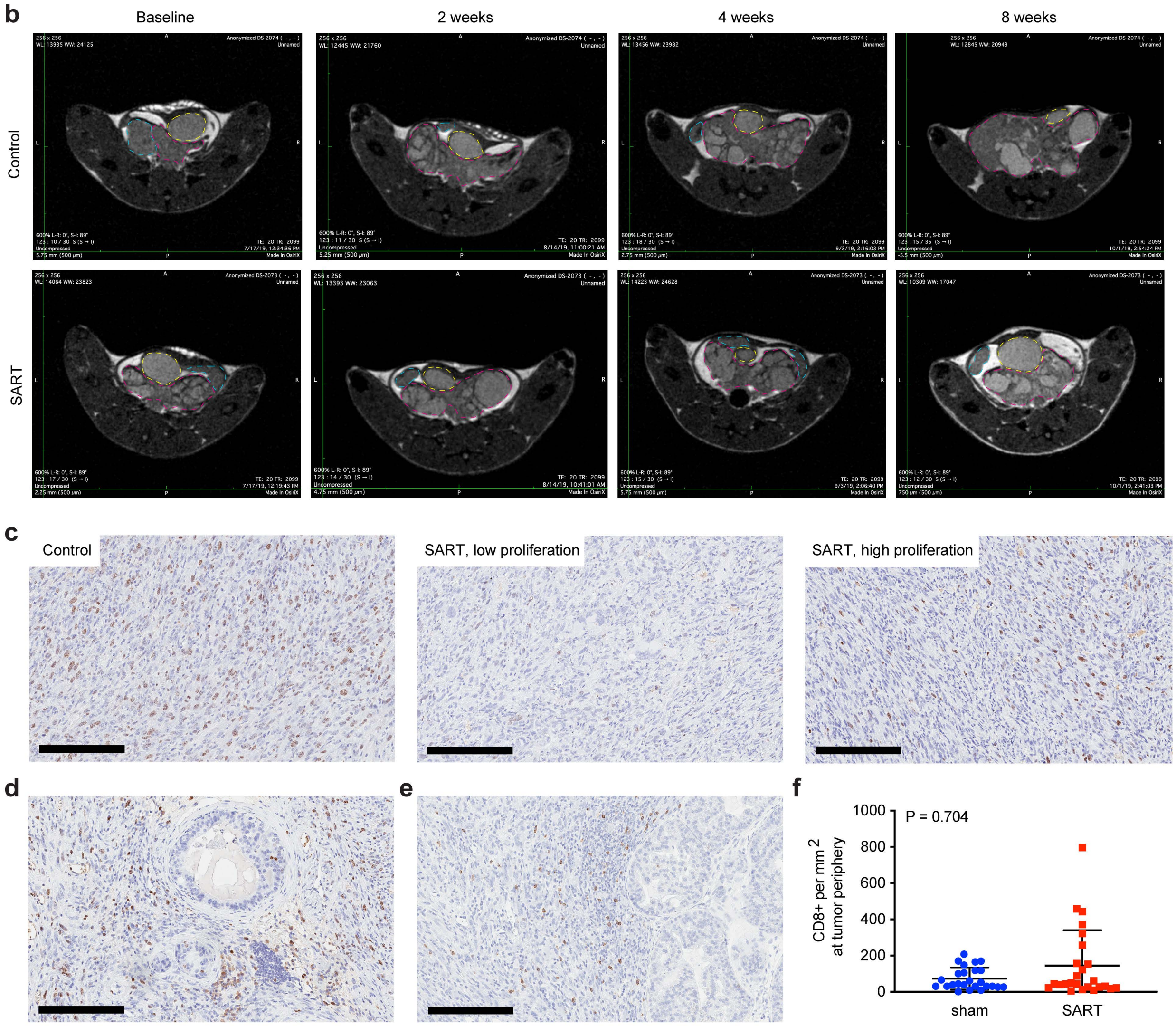

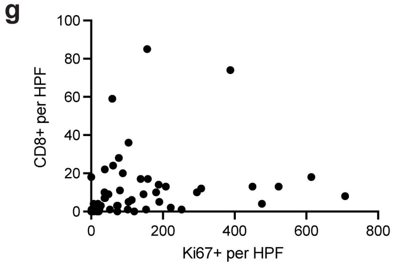

	Ki67+ per HPF vs. CD8+ per HPF
Spearman r	
r	0.3982
95% confidence interval	0.1265 to 0.6143
P value	
P (two-tailed)	0.0042

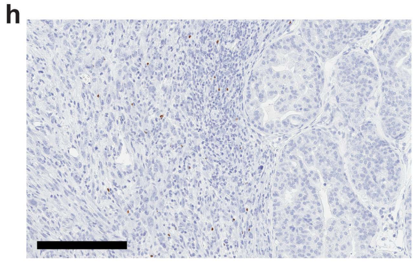

a Tumor progression in Study 1 assessed by serial MRI. Individual animal absolute volume data are shown. n=7 for sham (blue), n=14 for SART (red). **b** Representative axial slice of pelvic MRI at 4 months of age (baseline), and repeat MRI at 2, 4, and 8 weeks after treatment. Areas outlined are bladder (yellow), seminal vesicle (blue), prostate tumor (pink).

c Representative Ki67 IHC in an untreated tumor and two regions of a tumor 10 weeks post treatment with 5 fractions of 9 Gy. Scale bar = 200 μm. **d** Ki67 IHC in region of tumor with glands showing intraepithelial neoplasia and adjacent lymphocytic infiltrates. Scale bar = 200 μm. **e** Representative CD8 IHC in an untreated tumor. Scale bar = 200 μm. **f** CD8+ T cells near the tumor periphery assessed by IHC in SART-treated tumors and controls as in Fig. 6m, 5 HPF per tumor, 5 tumors per treatment group. Mean +/- SD. P value estimate by Mann-Whitney test. **g** CD8+ and Ki67+ cells were quantified in the same regions of 5 treated tumors (10 HPF per tumor) and correlation estimated as shown. **h** Representative FOXP3 IHC in an untreated tumor. Scale bar = 200 μm.

Supplementary Fig. 11: Evaluating stereotactic ablative radiotherapy (SART) effects in a mouse colorectal adenoma model.

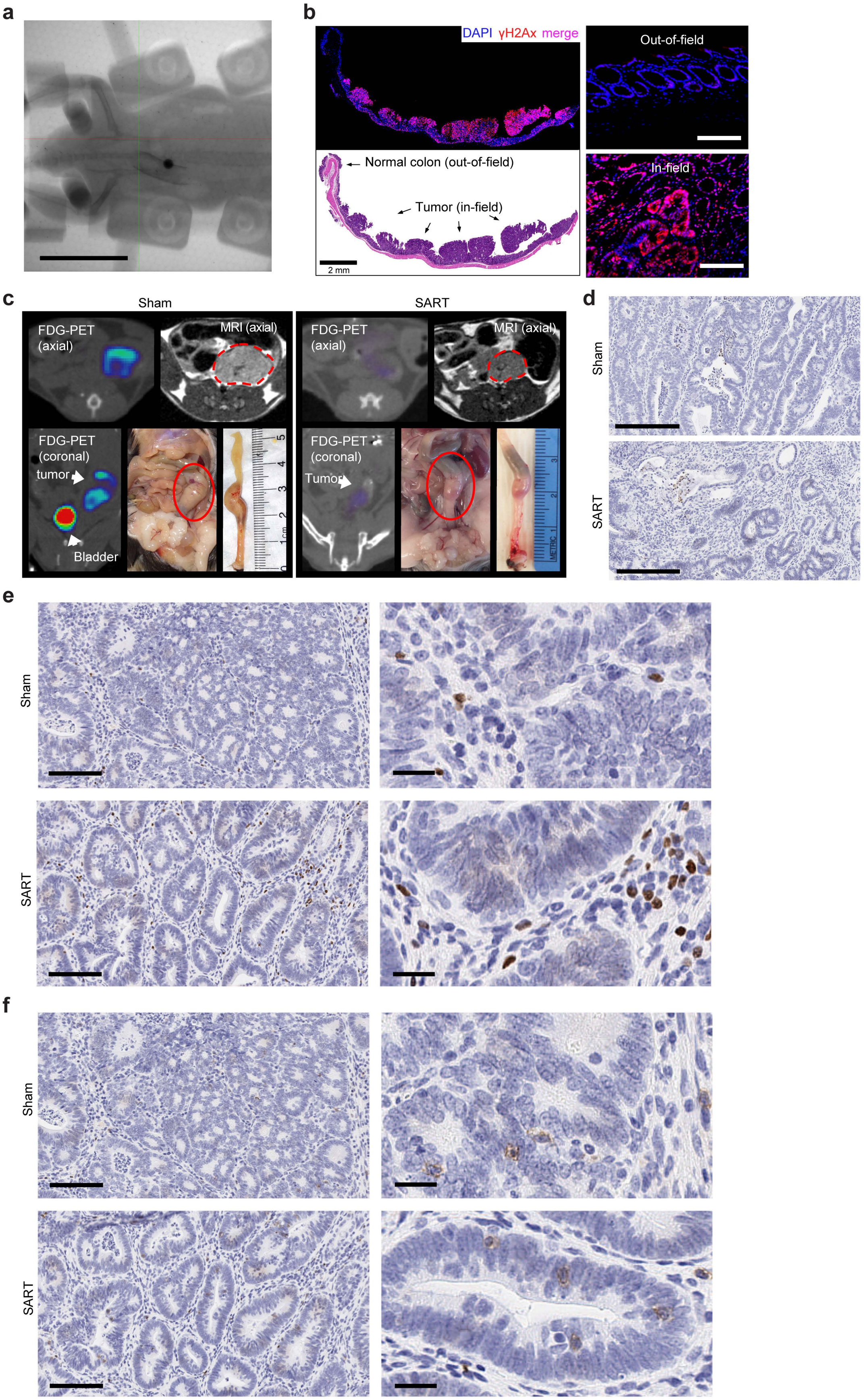

a Under endoscopic guidance 200 μL radiopaque contrast was injected into the submucosa 2-3 cm from the anal verge to identify the region of rectum at risk for tumor induction. The animal was then imaged in the restrainer with a radiopaque marker on the restrainer at center of the treatment field. The representative fluoroscopic image shows good placement of

the rectal tumor target region relative to the treatment isocenter. Scale bar = 2 cm. **b** Evaluation of focal DNA damage in a longitudinal cross-section of colorectum by γ-H2AX IHC. This mouse with multifocal adenoma was treated with 7.5 Gy to a 2 cm diameter radiation field 30 minutes prior to tissue collection. Nuclear γ-H2AX foci are seen only in the portion of the colorectum that was within the radiation field. Uniform DNA damage induction is seen throughout the entire tumor and small margin of adjacent normal tissue. Scale bar = 2 mm for the low power images and 0.1 mm for the high-power views. **c** FDG-PET, MRI, and gross pathology of a representative SART-treated animal 6 months after treatment with 7.5 Gy x 5 fractions QOD. Untreated control is shown for comparison. **d** Representative cleaved caspase-3 IHC images of tumors from study 2. Scale bar = 200 μm. **e** Representative FOXP3 IHC images of tumors from study 2. Scale bar = 100 μm (low power view), 20 μm (high power view). f Representative CD8 IHC images of tumors from study 2. Scale bar = 100 μm (low-power view), 20 μm (high-power view).

SUPPLEMENTARY METHODS

This supplementary methods section contains blueprints (to scale) for the lead shields, collimators, and mouse restrainers used to deliver stereotactic radiotherapy in this study. There are two different restrainers designed for use with animals of the body weight indicated. The measurements for each restrainer are shown separately for US and metric units. Also included are instructions for restrainer assembly and use.

Restrainer for 24-34 g mouse
(drawn to scale)

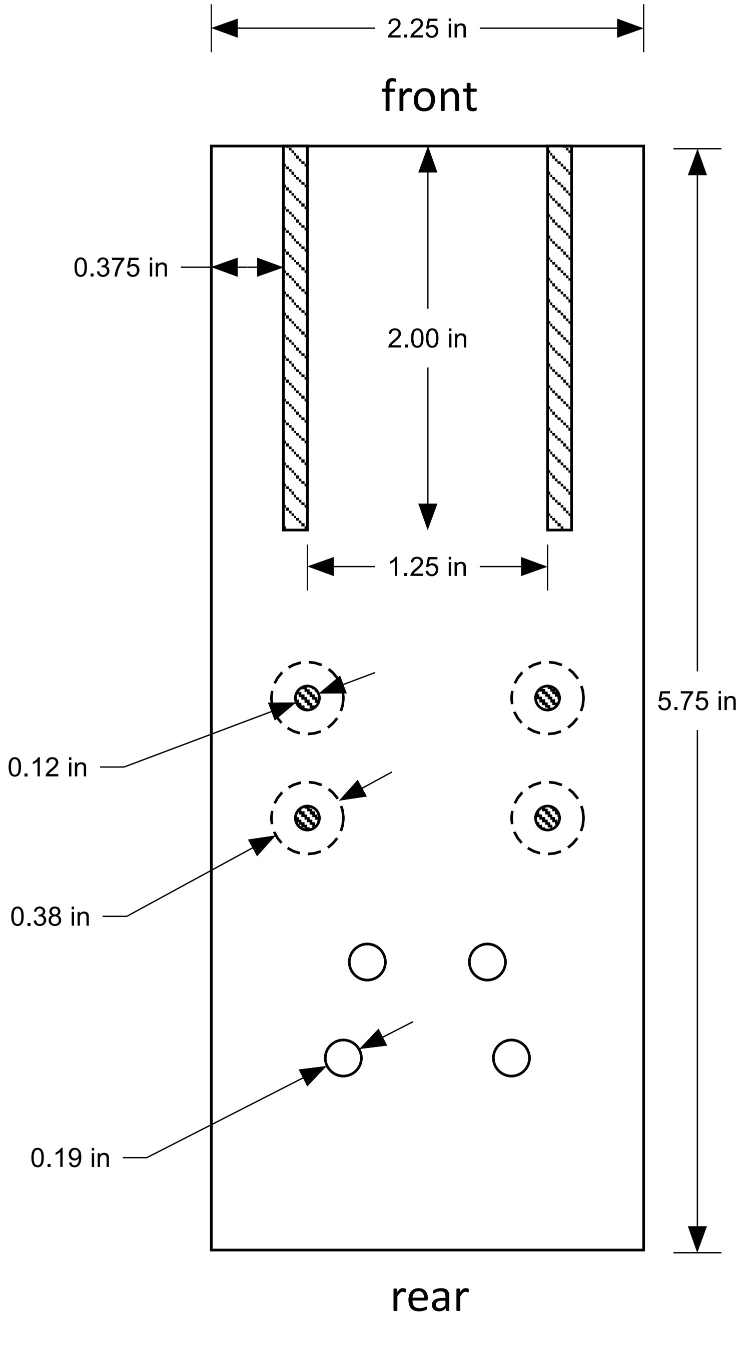

bottom view

Restrainer for 24-34 g mouse
(drawn to scale)

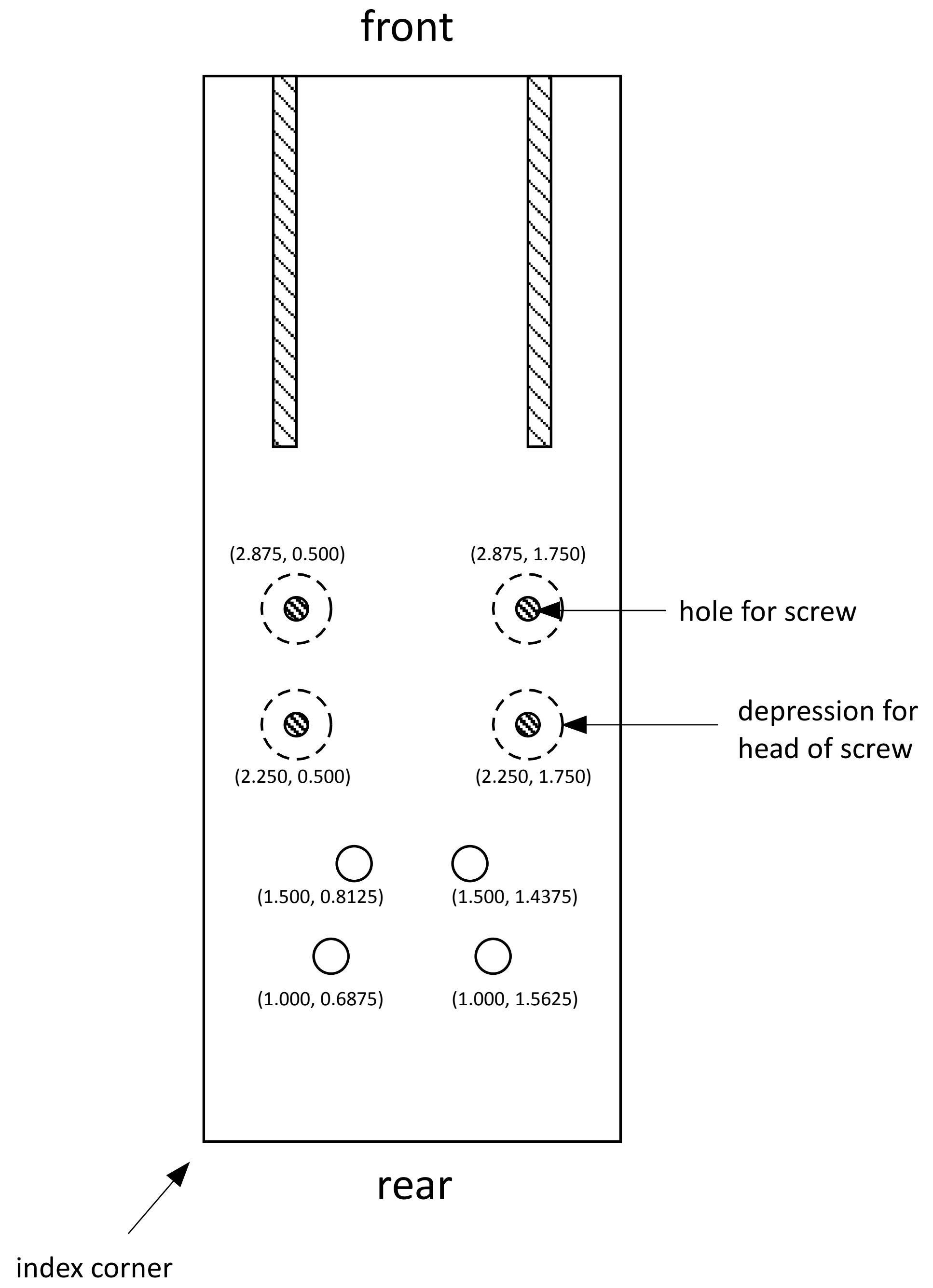

numbers in parentheses show distance (in.) from center (vertical, horizontal) to index corner

bottom view

Restrainer for 24-34 g mouse
(drawn to scale)

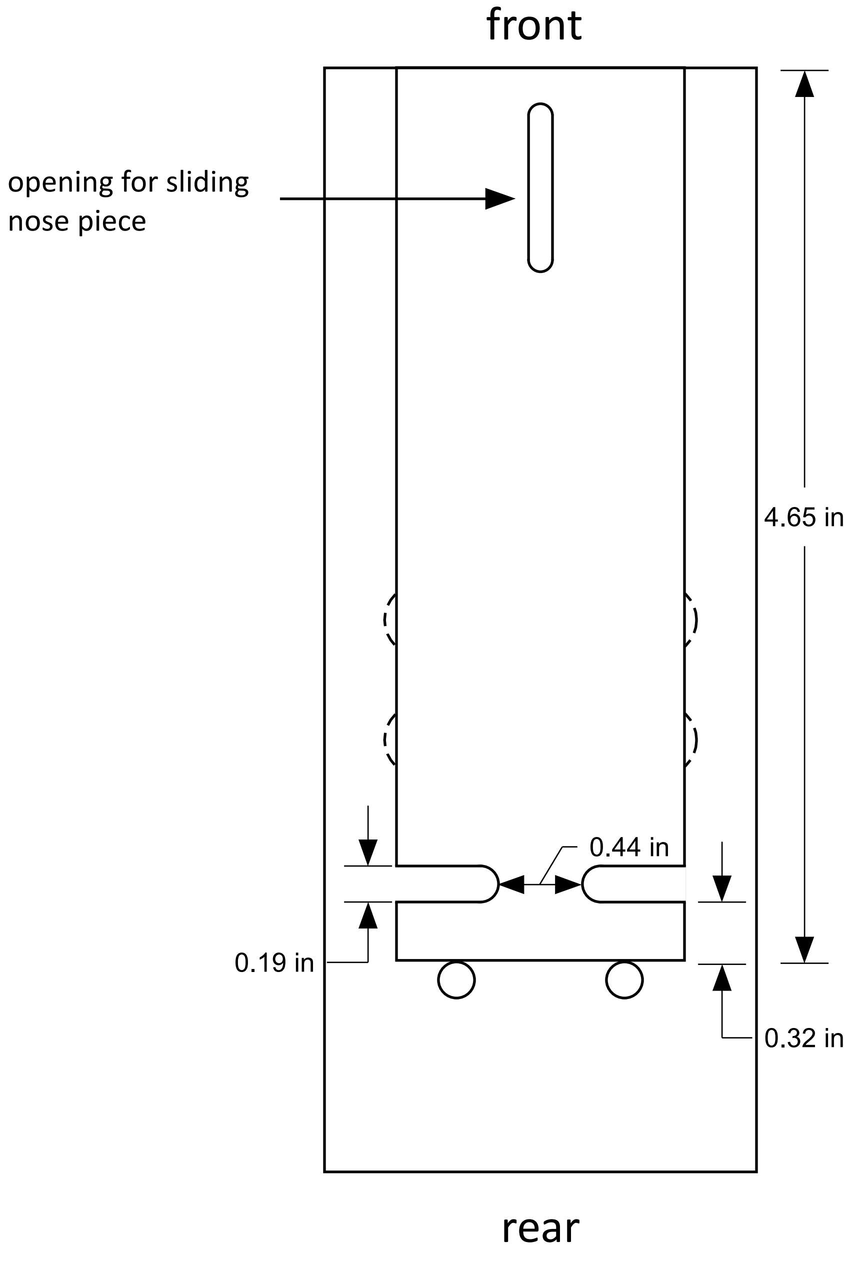

top view

Restrainer for 24-34 g mouse
(drawn to scale)

side view

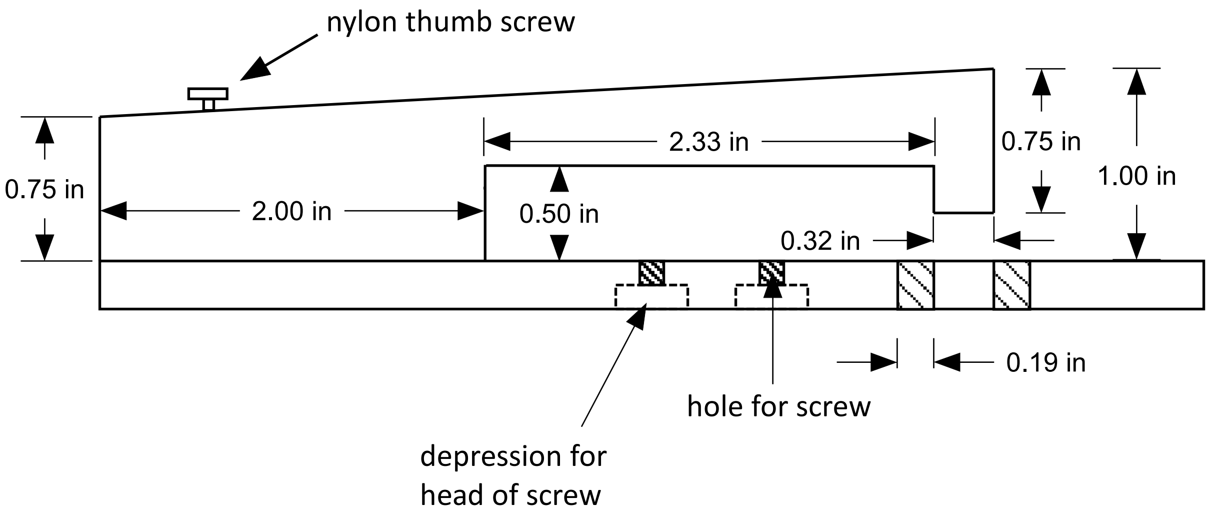

front view

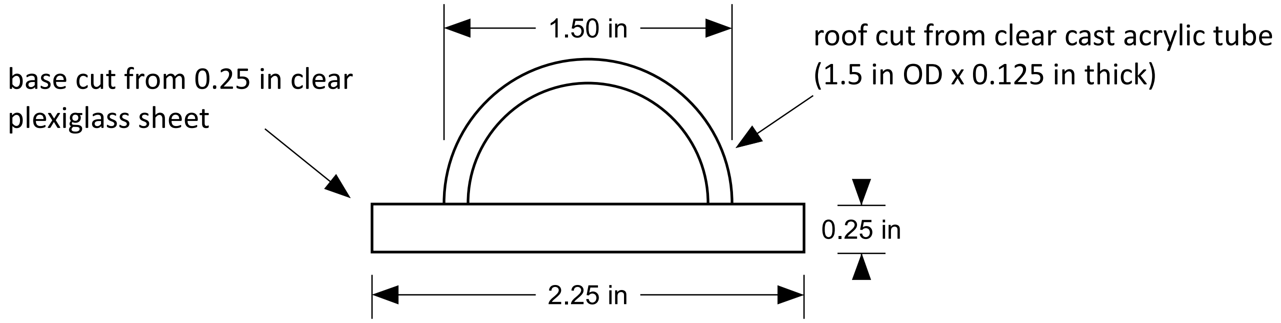

Restrainer for 24-34 g mouse
(drawn to scale)

nose piece

cut from acrylic rod

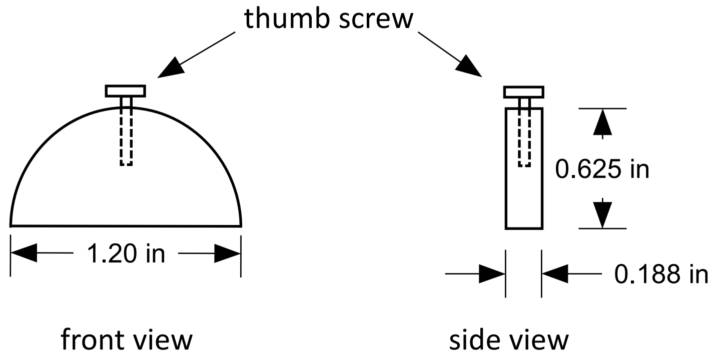

hind limb pins

cut from acrylic rod

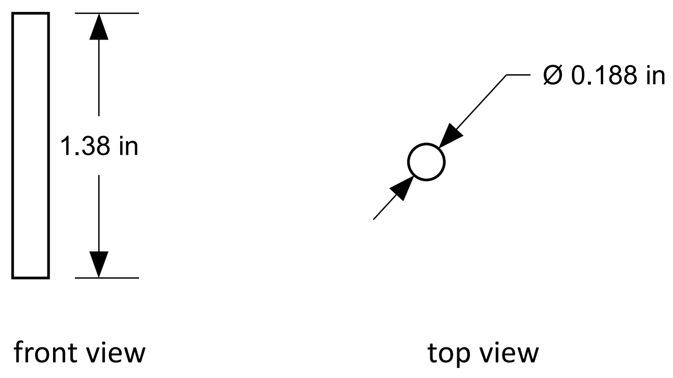

Restrainer for 24-34 g mouse
(drawn to scale)

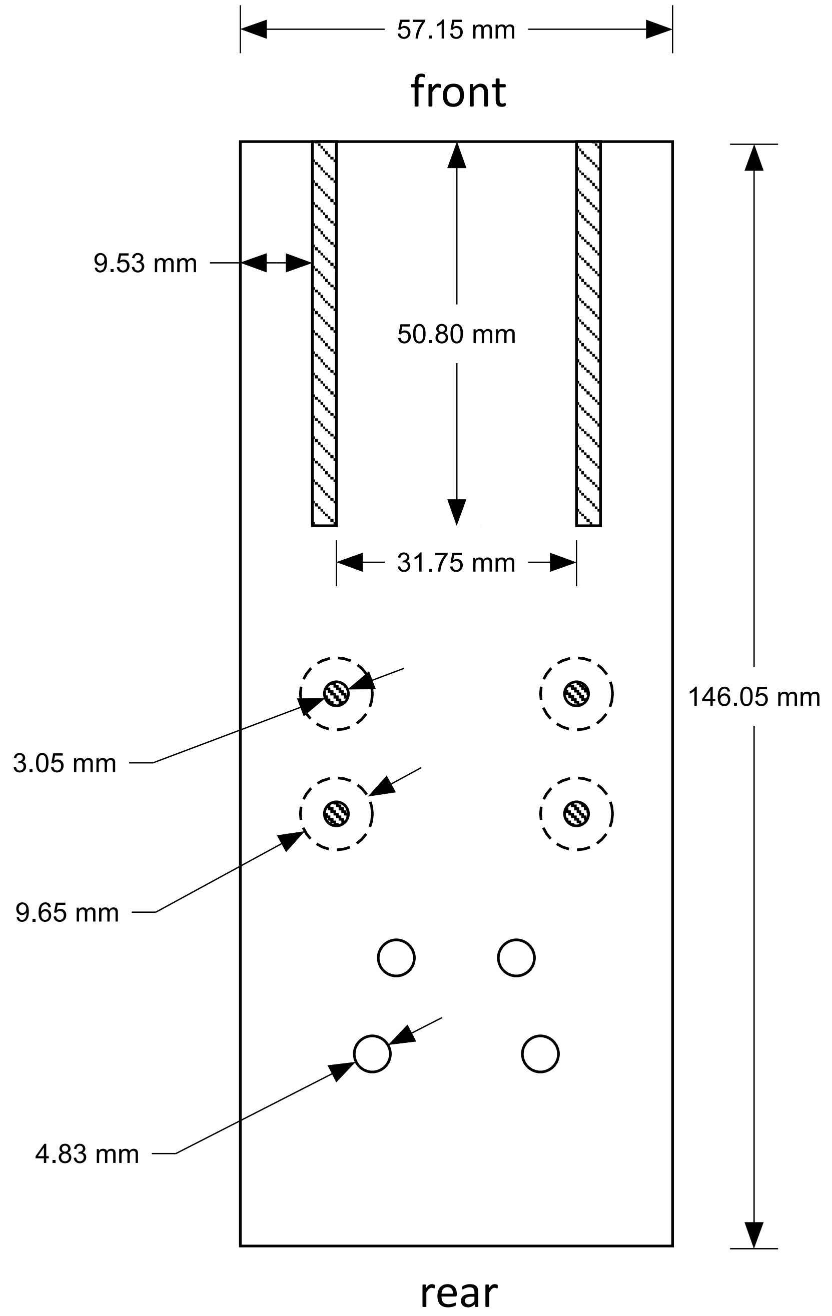

bottom view

Restrainer for 24-34 g mouse
(drawn to scale)

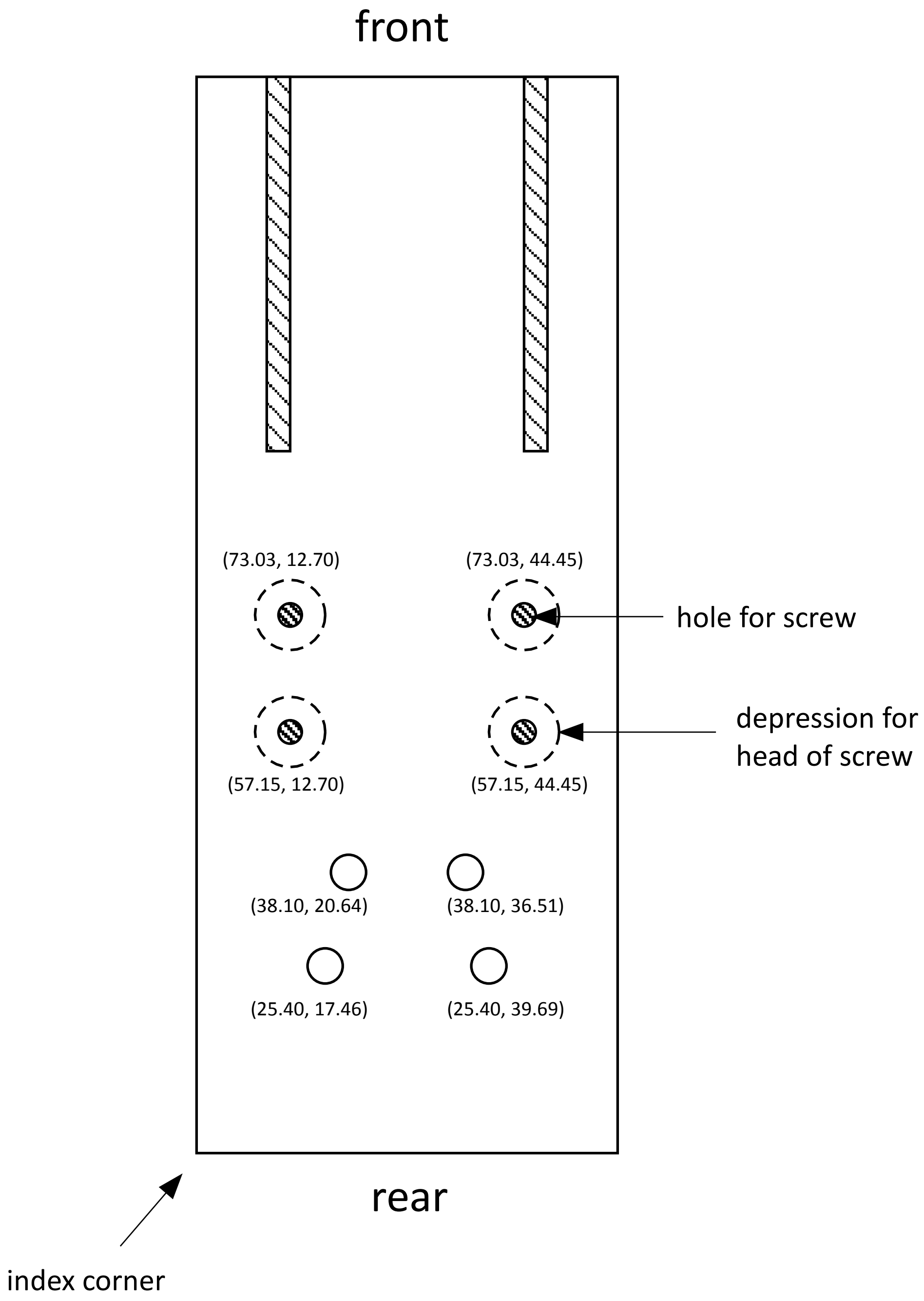

numbers in parentheses show distance (mm) from center (vertical, horizontal) to index corner

bottom view

Restrainer for 24-34 g mouse
(drawn to scale)

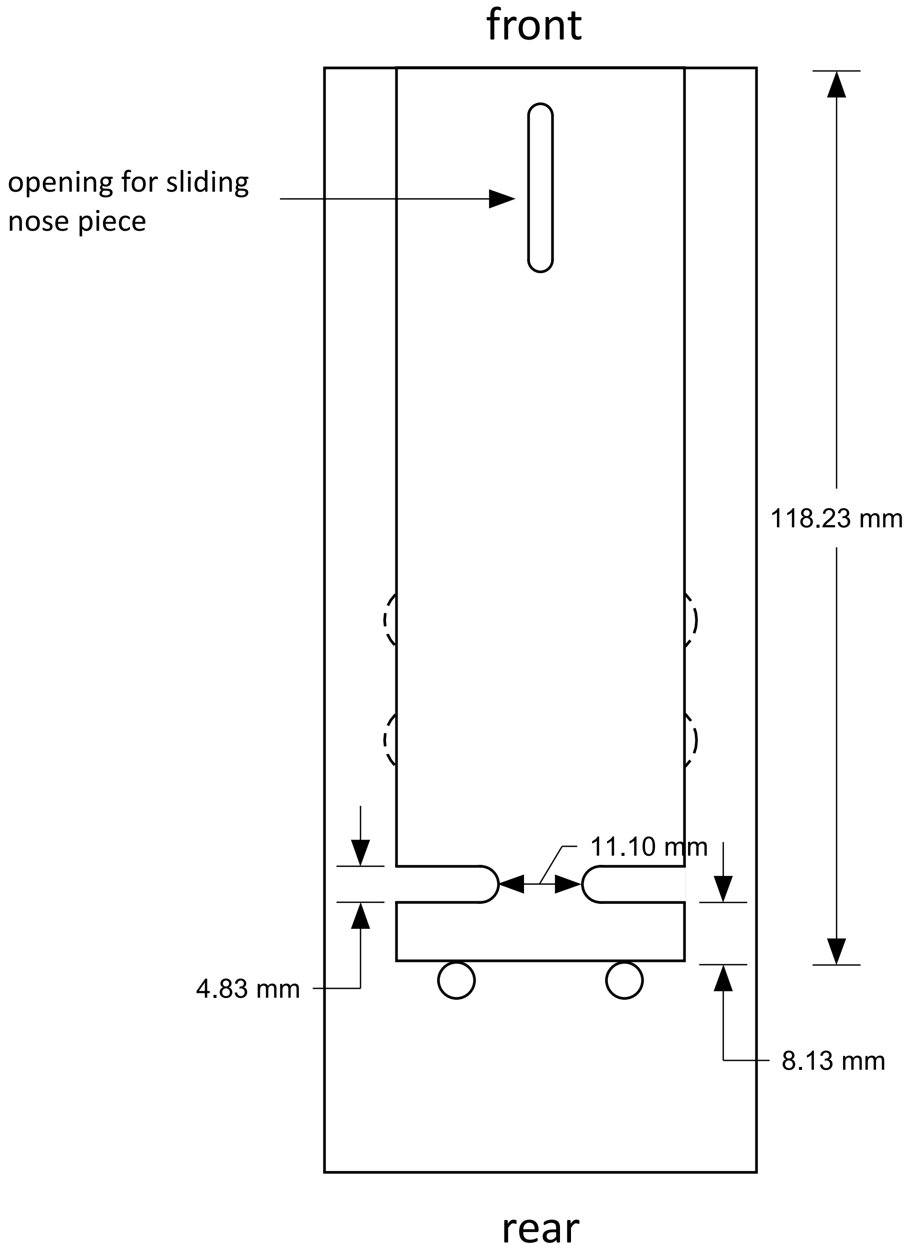

top view

Restrainer for 24-34 g mouse
(drawn to scale)

side view

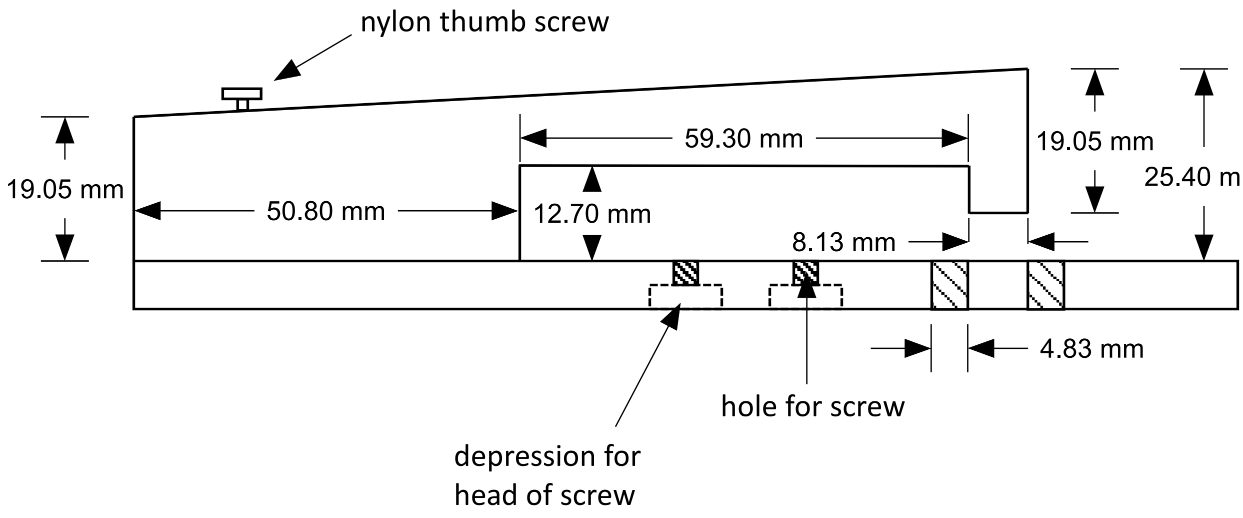

front view

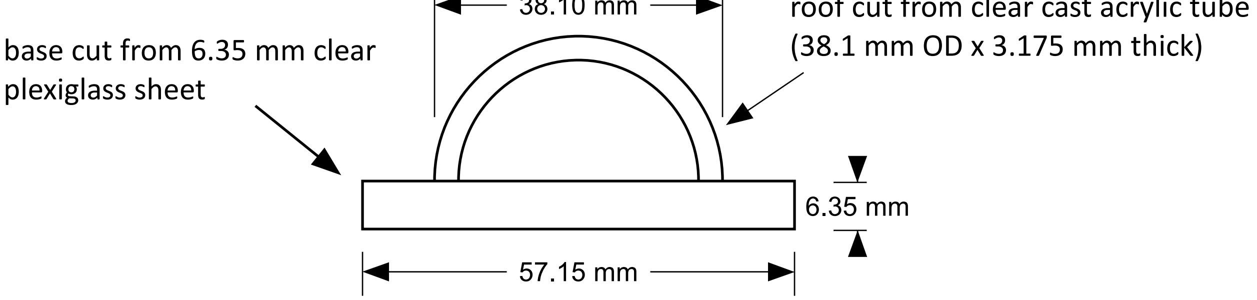

Restrainer for 24-34 g mouse (drawn to scale)

nose piece

cut from acrylic rod

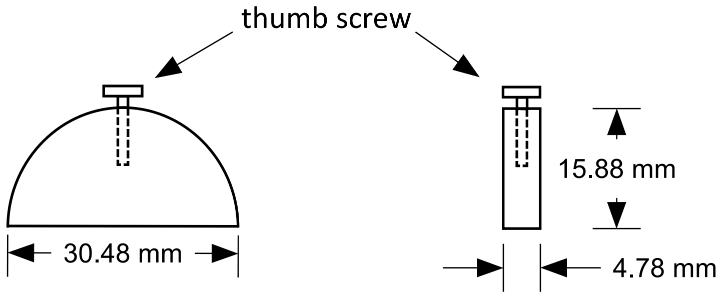

front view

side view

hind limb pins

cut from acrylic rod

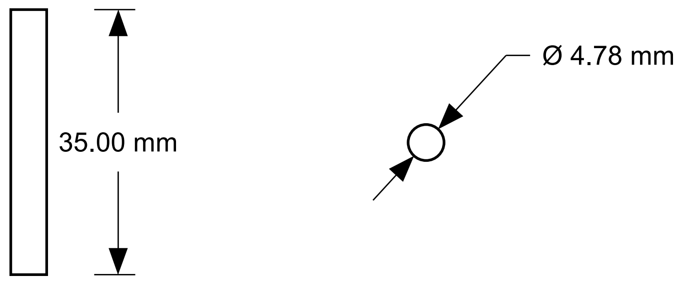

front view

top view

Restrainer for 35-47 g mouse
(drawn to scale)

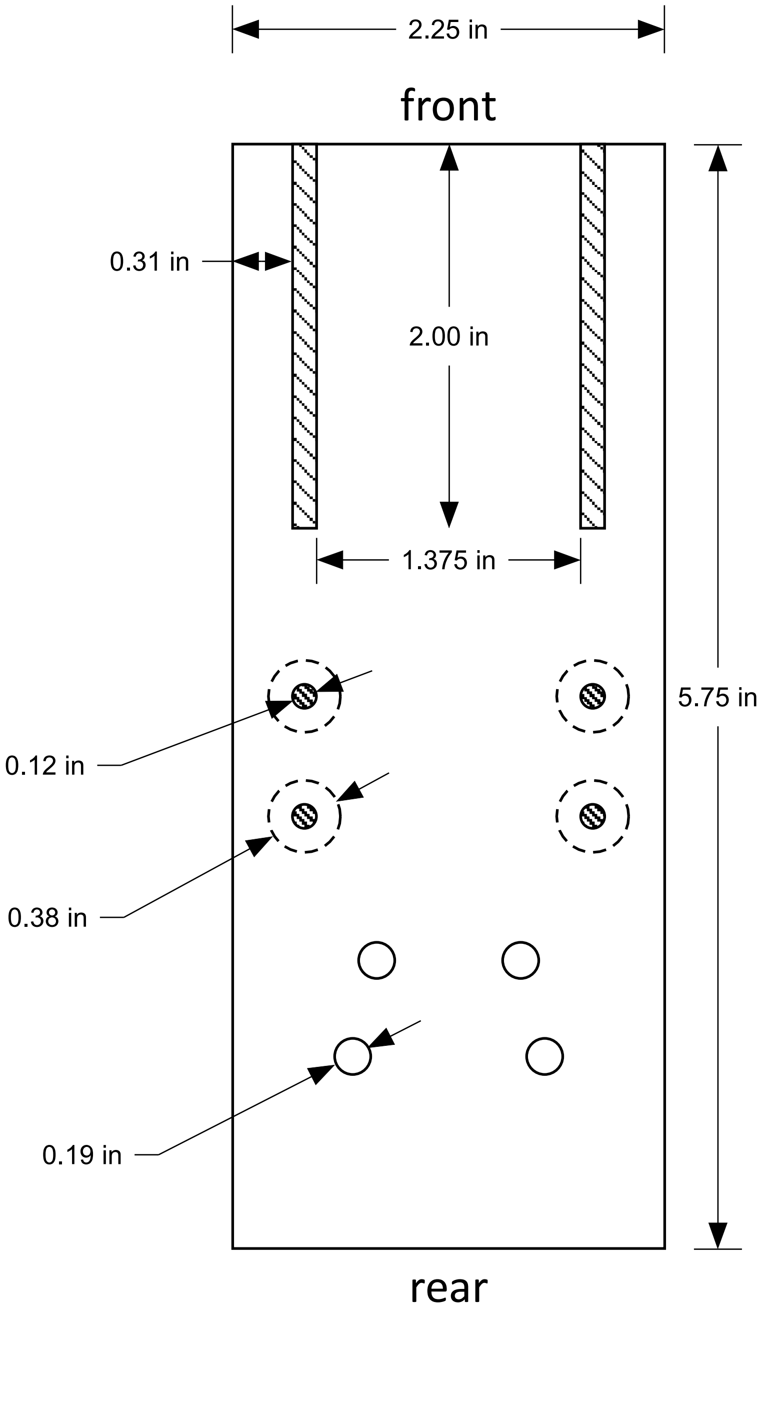

bottom view

Restrainer for 35-47 g mouse
(drawn to scale)

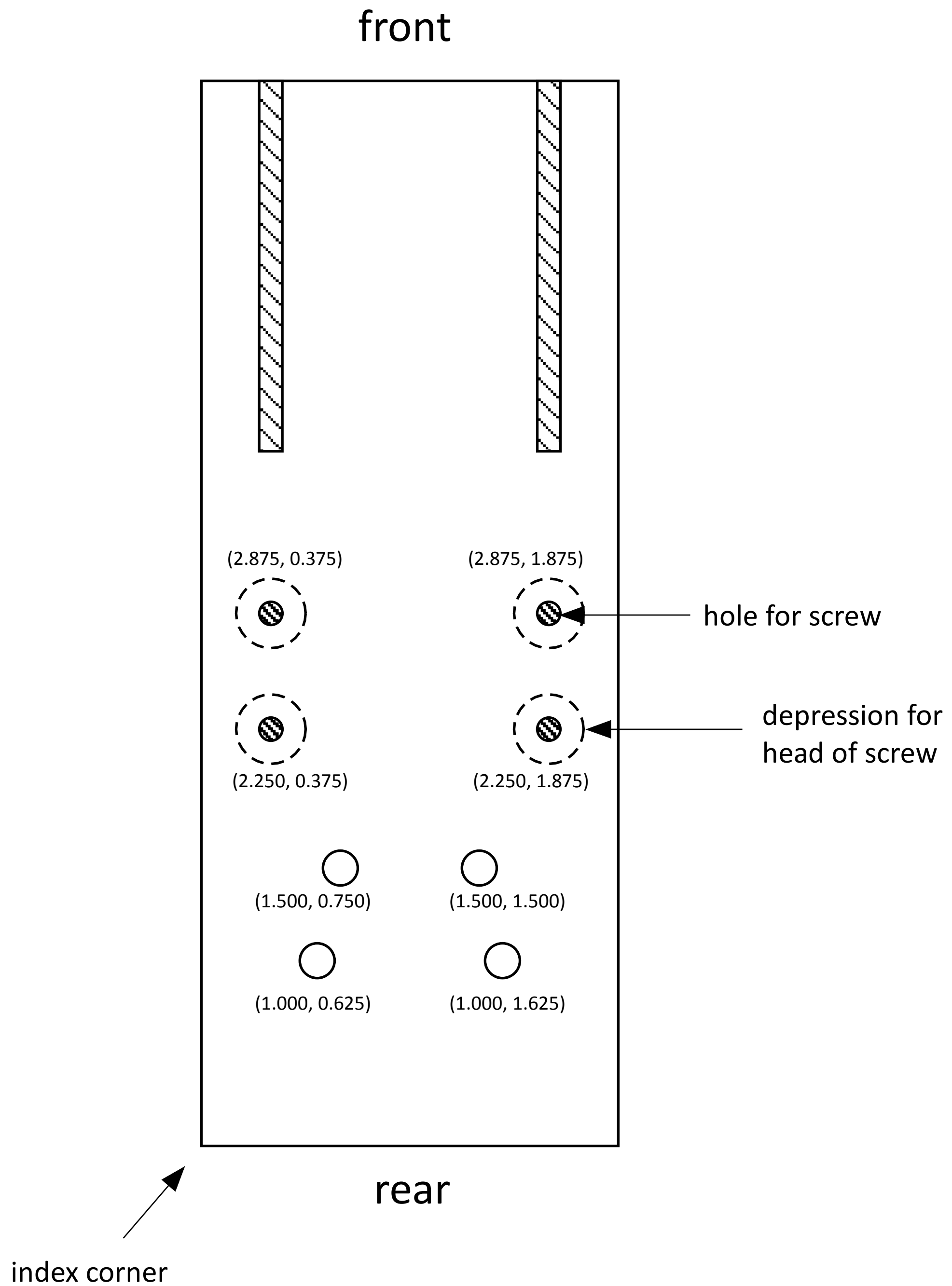

numbers in parentheses show distance (in.) from center (vertical, horizontal) to index corner

bottom view

Restrainer for 35-47 g mouse
(drawn to scale)

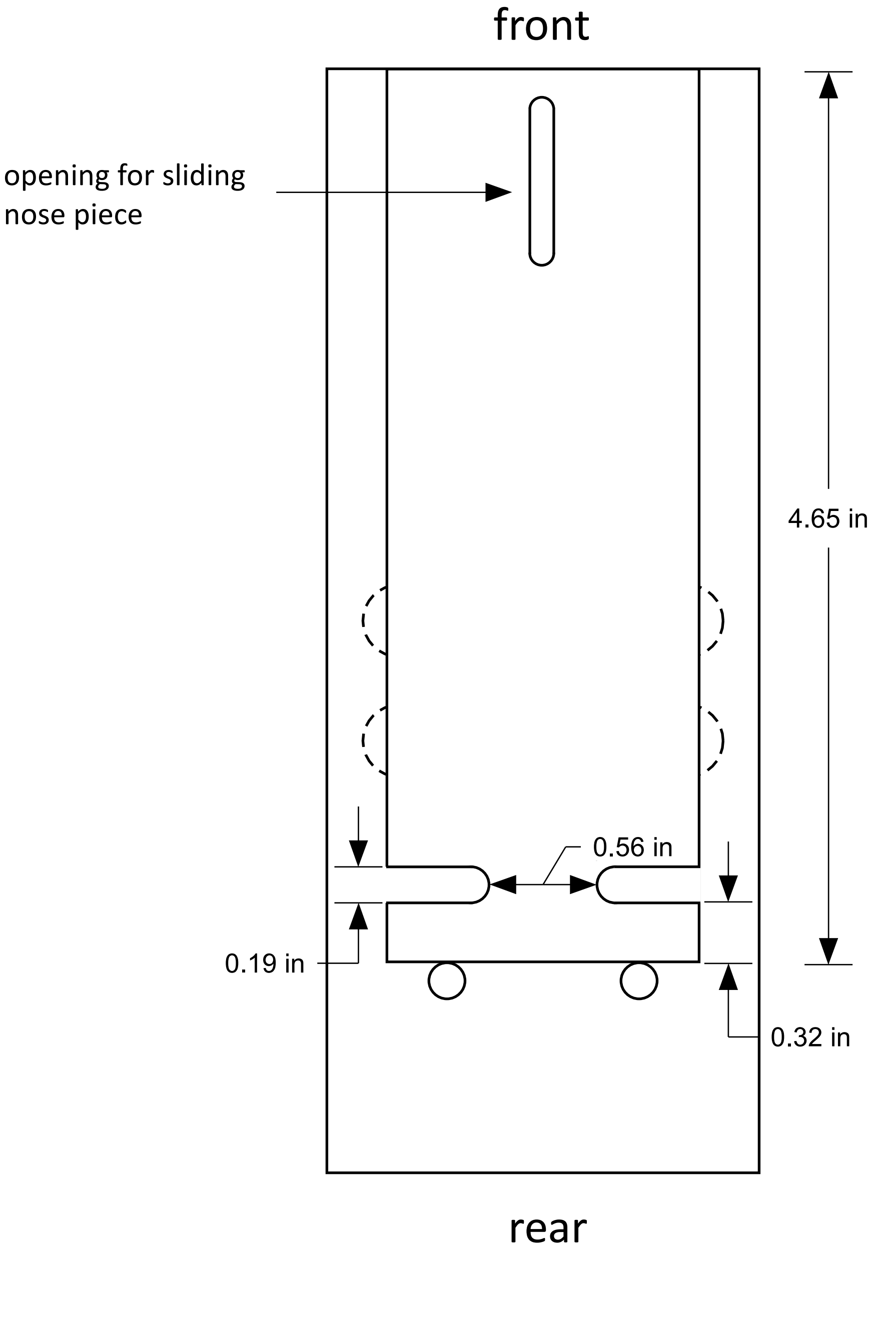

top view

Restrainer for 35-47 g mouse
(drawn to scale)

side view

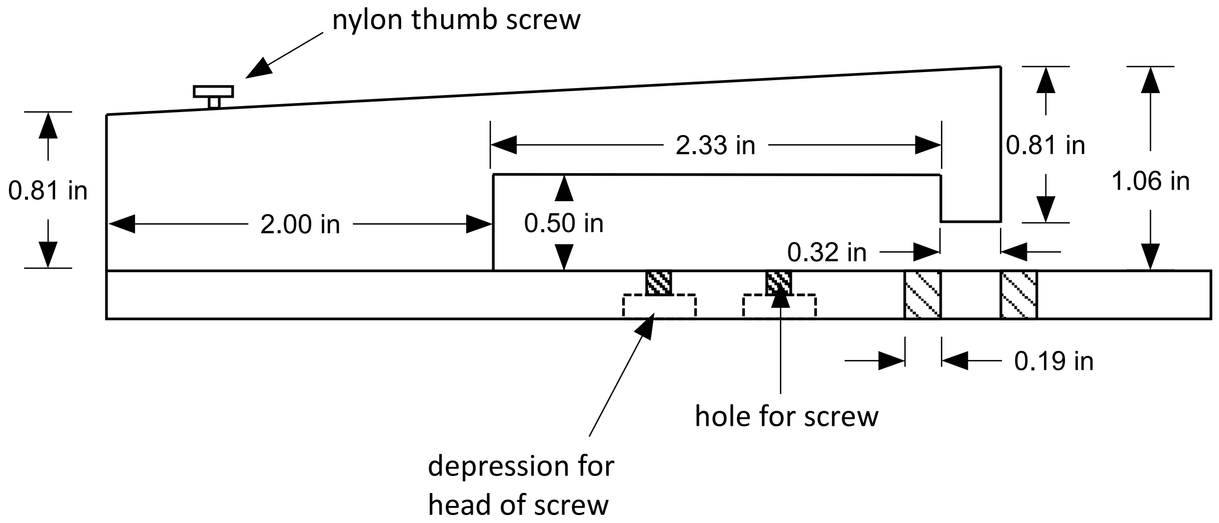

front view

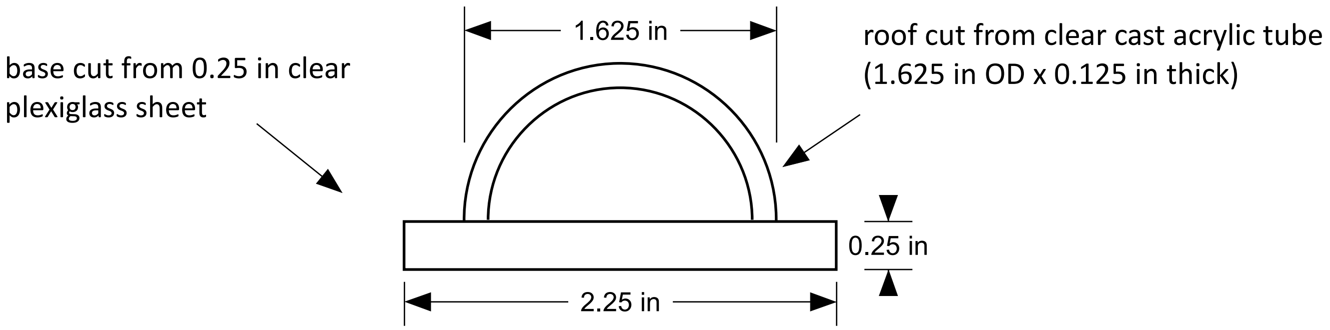

Restrainer for 35-47 g mouse
(drawn to scale)

nose piece

cut from acrylic rod

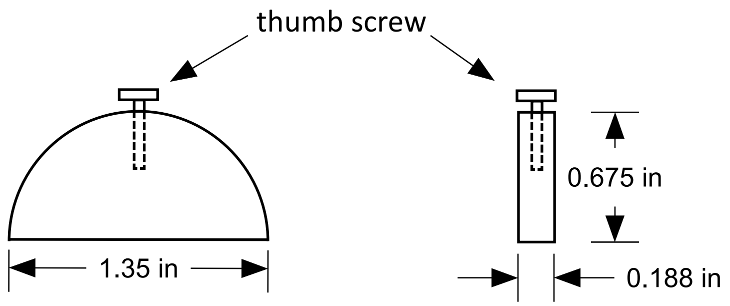

front view side view

hind limb pins

cut from acrylic rod

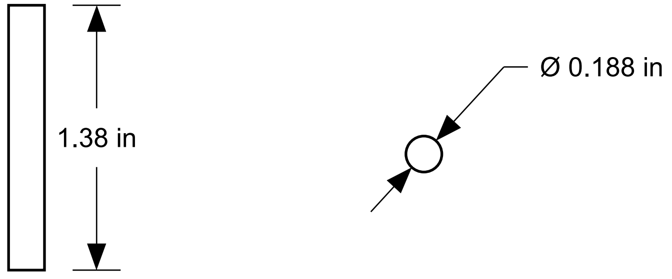

front view top view

Restrainer for 35-47 g mouse
(drawn to scale)

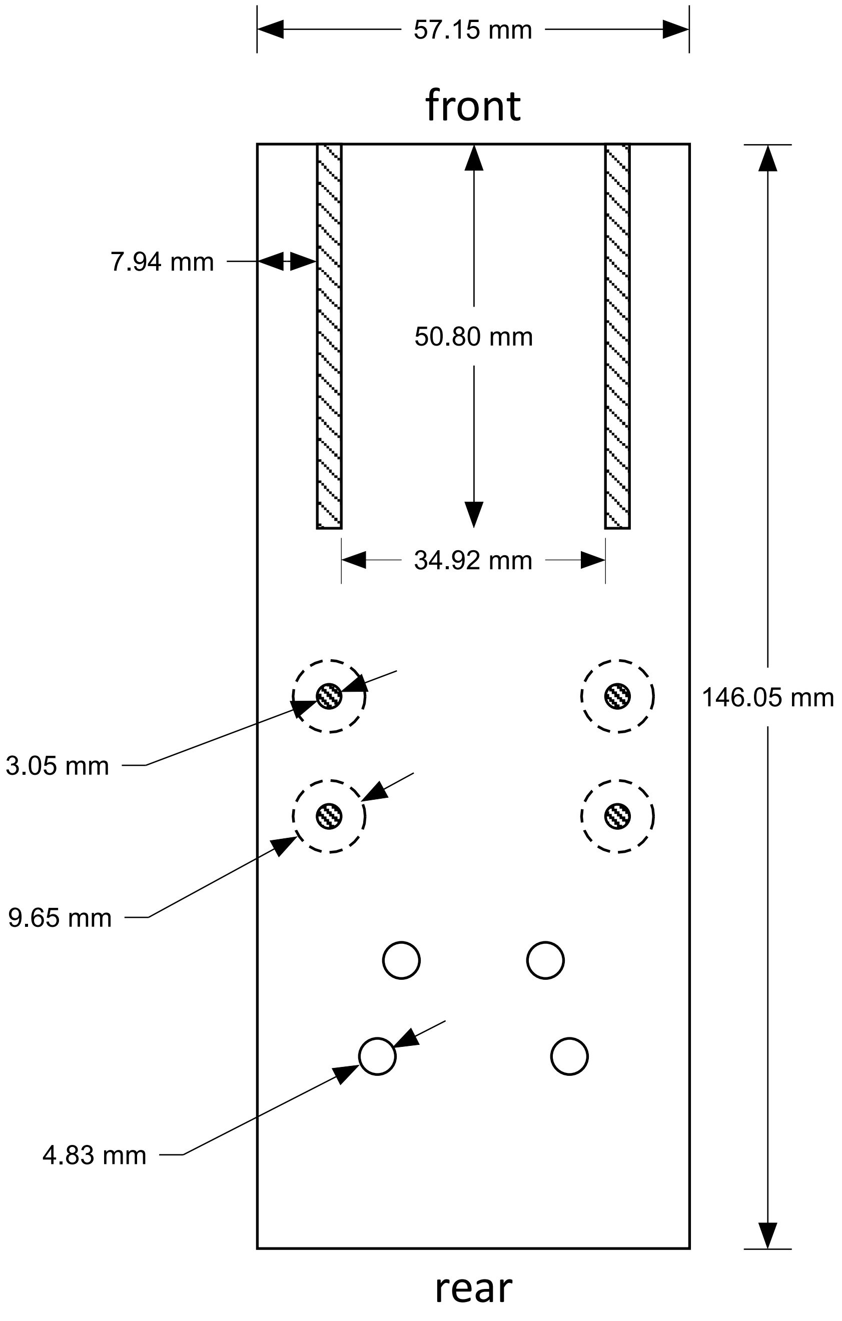

bottom view

Restrainer for 35-47 g mouse
(drawn to scale)

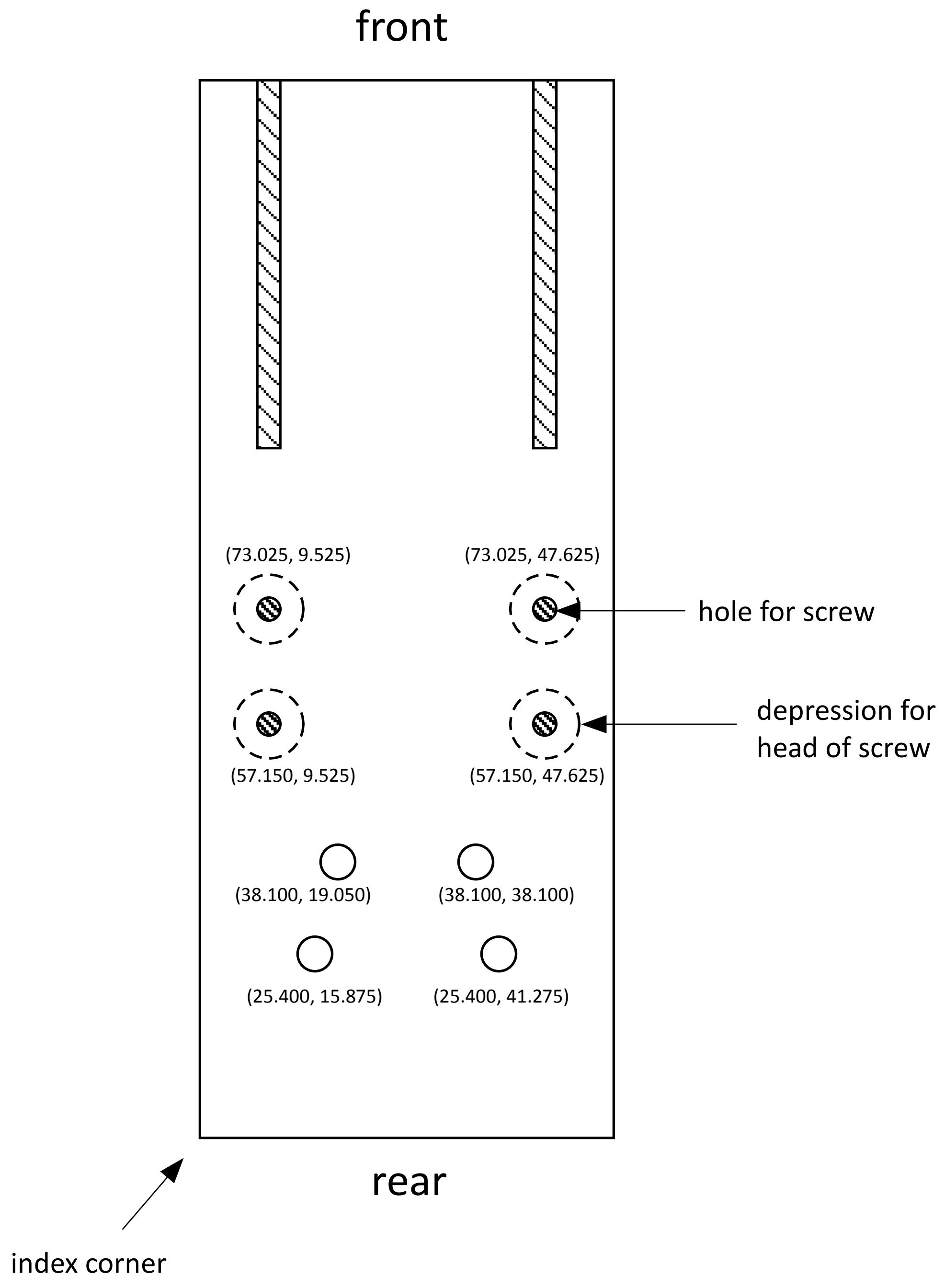

numbers in parentheses show distance (mm) from center (vertical, horizontal) to index corner

bottom view

Restrainer for 35-47 g mouse
(drawn to scale)

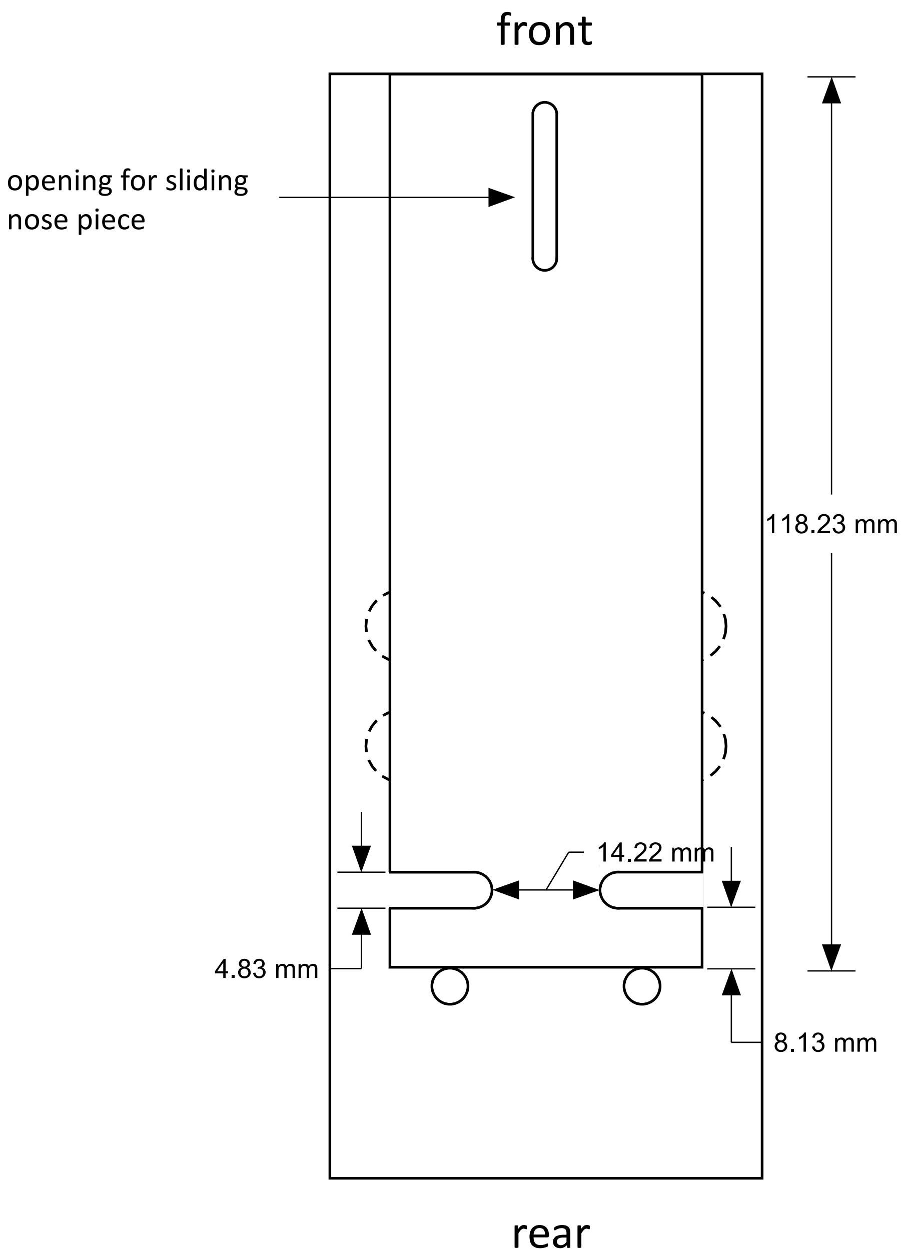

top view

Restrainer for 35-47 g mouse
(drawn to scale)

side view

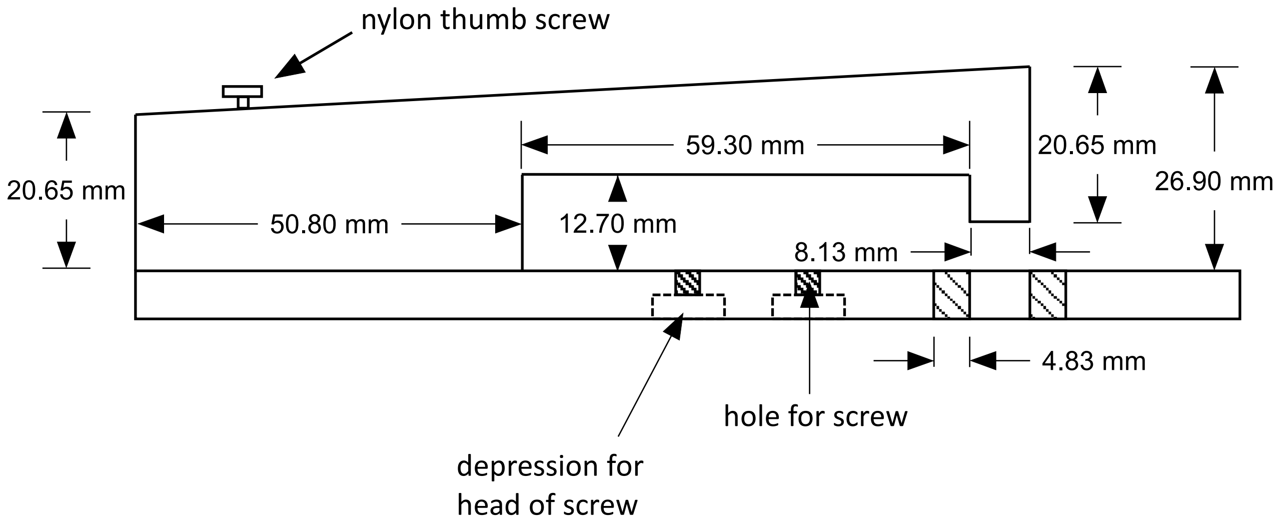

front view

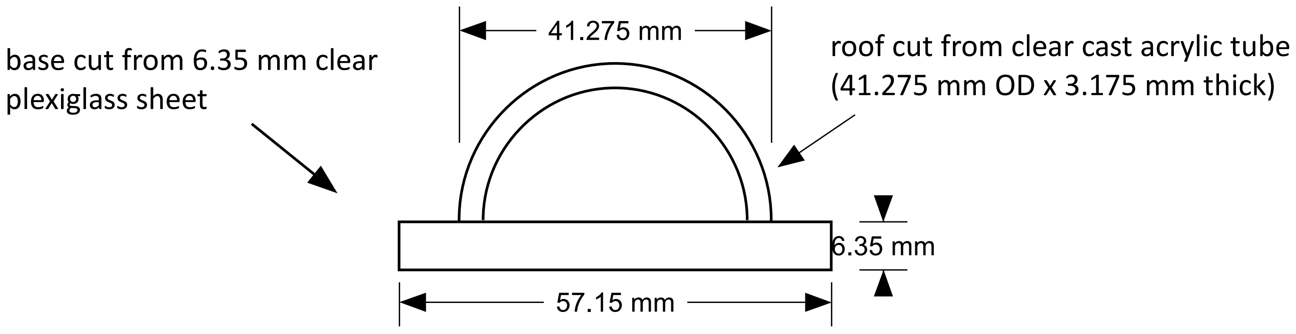

Restrainer for 35-47 g mouse
(drawn to scale)

nose piece

cut from acrylic rod

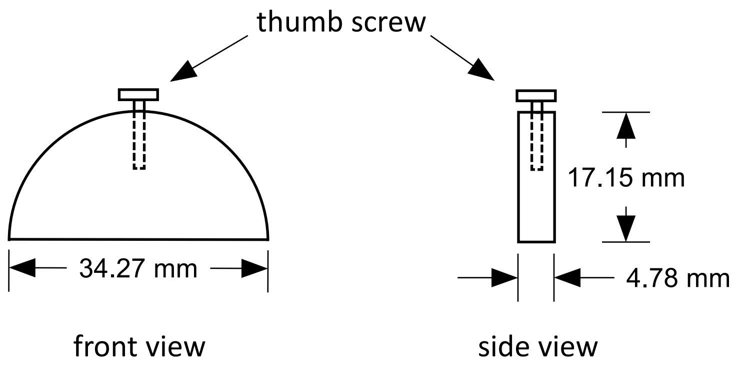

hind limb pins

cut from acrylic rod

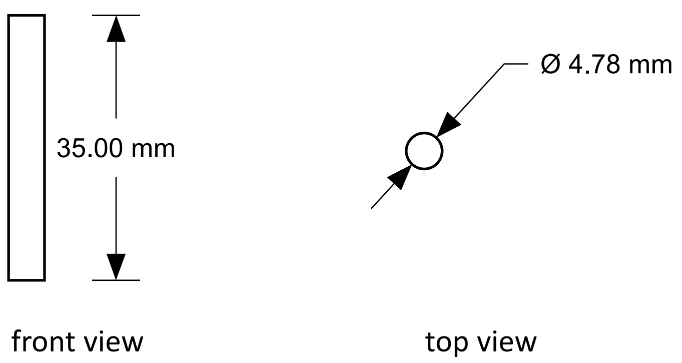

Universal side blocks
(drawn to scale)

thorax blocks

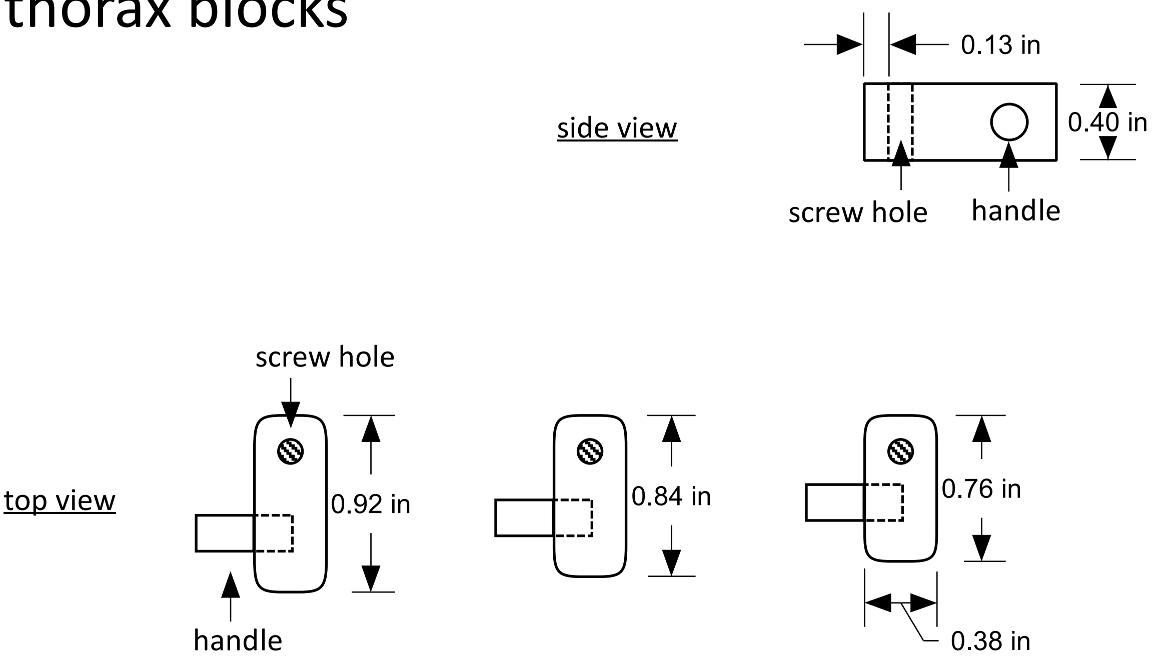

abdominal-pelvic blocks

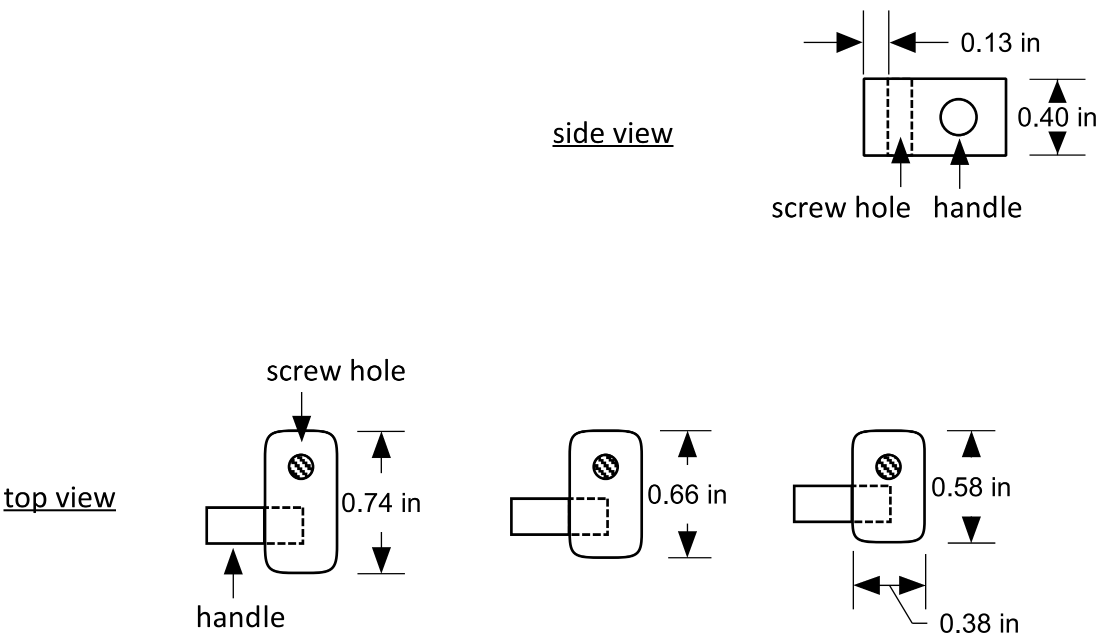

Universal side blocks
(drawn to scale)

thorax blocks

<u>side view</u>

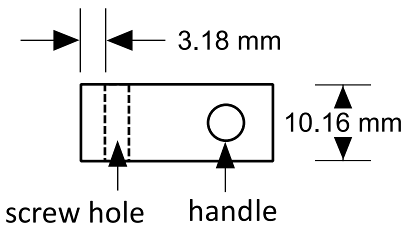

<u>top view</u>

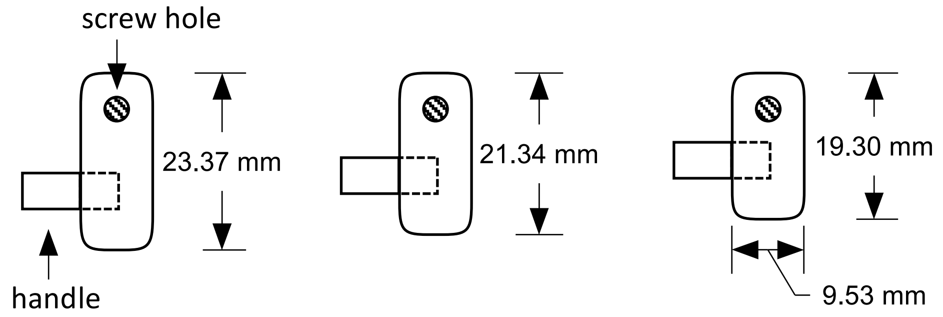

abdominal-pelvic blocks

<u>side view</u>

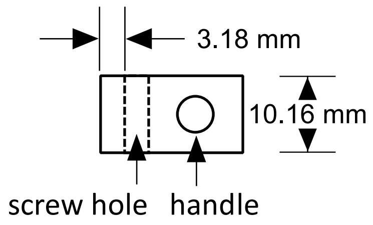

<u>top view</u>

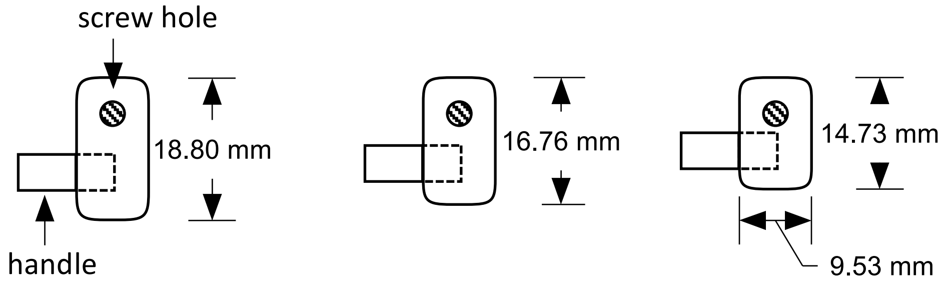

Restrainer assembly and use instructions

Step 2: Anterior set of hind limb pins are inserted medial to femur to prevent animal from exiting the chamber.

Step 6: Sliding nose piece is adjusted to mouse length and secured by tightening thumb screw.
(note: animal is now secured)

Step 4: Posterior set of hind limb pins are inserted lateral to the ankle.
(note: the knee joint is now secured)

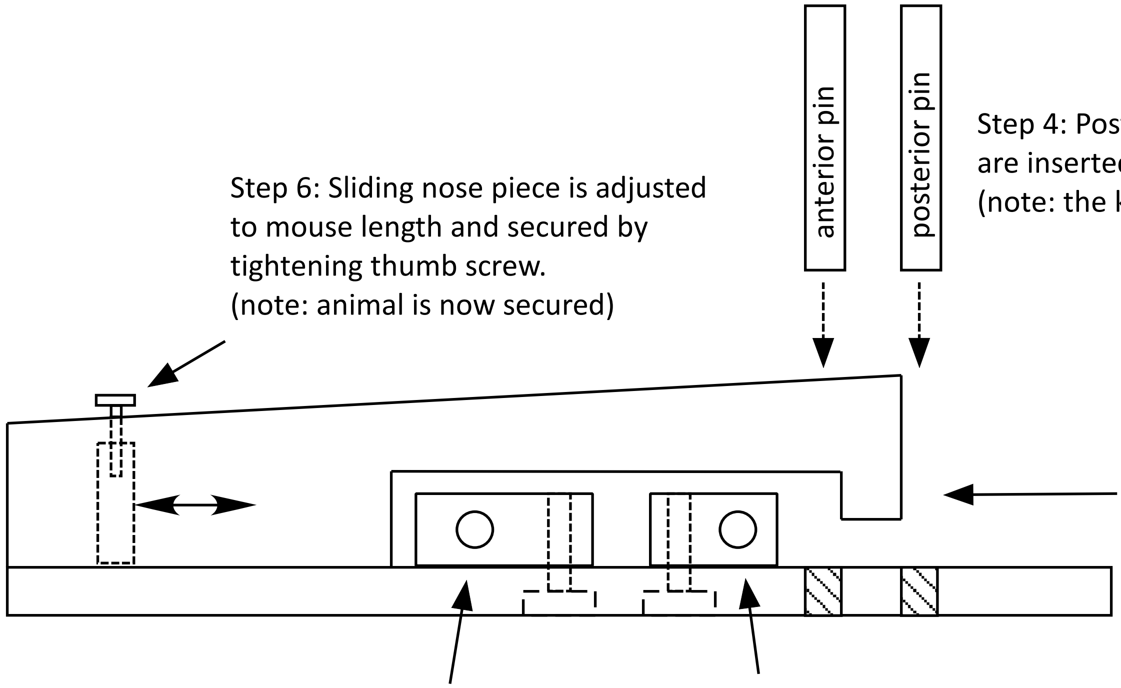

Step 1: Animal enters chamber head-first from rear of holder in prone ("belly down") position.

Step 5: Thorax blocks are adjusted to secure forelimbs into a forward ("arms up") position.

Step 3: Hind limbs are brought into caudal ("legs extended") position and secured by adjusting abdominopelvic blocks.
(note: the femur is now secured)

(note: thorax and abdominopelvic blocks are secured by tightening nylon screws on underside of holder)

Restrainer assembly and use instructions

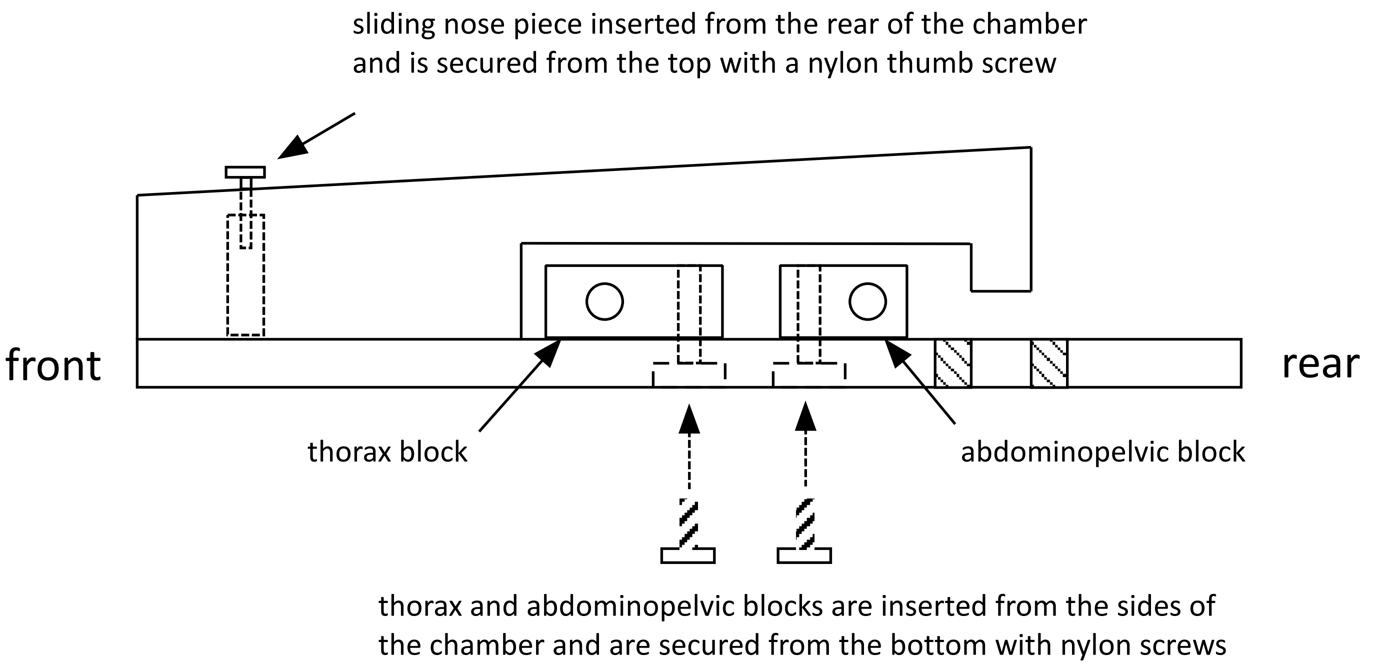

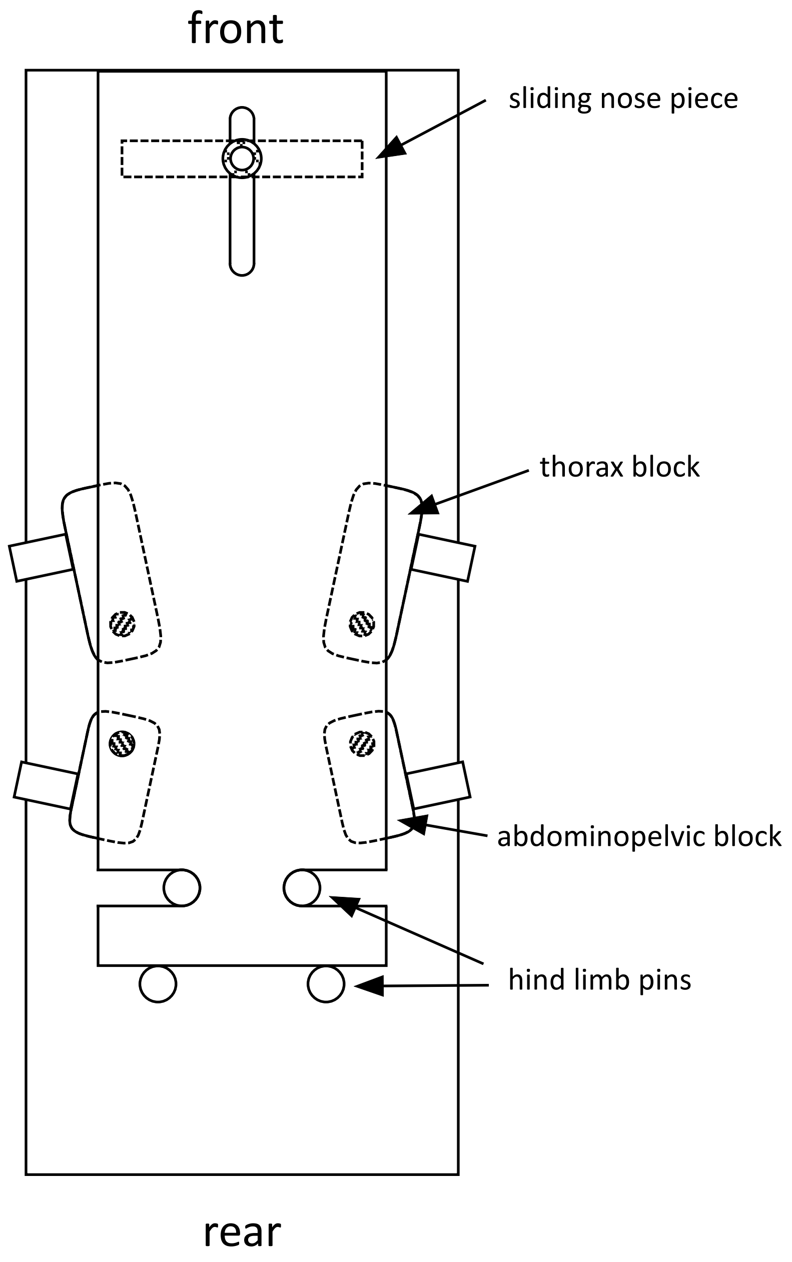

table of recommended block size by mouse weight				
mouse weight	thoracic block length		abdominopelvic block length	
(grams)	(inches)	(mm)	(inches)	(mm)
24 - 25	0.760	19.3	0.740	18.8
26 - 28	0.760	19.3	0.660	16.8
29 - 32	0.840	21.3	0.660	16.8
33 - 40	0.840	21.3	0.580	14.7
41 - 47	0.920	23.4	0.580	14.7

Lead shield
(drawn at 1:2 scale)

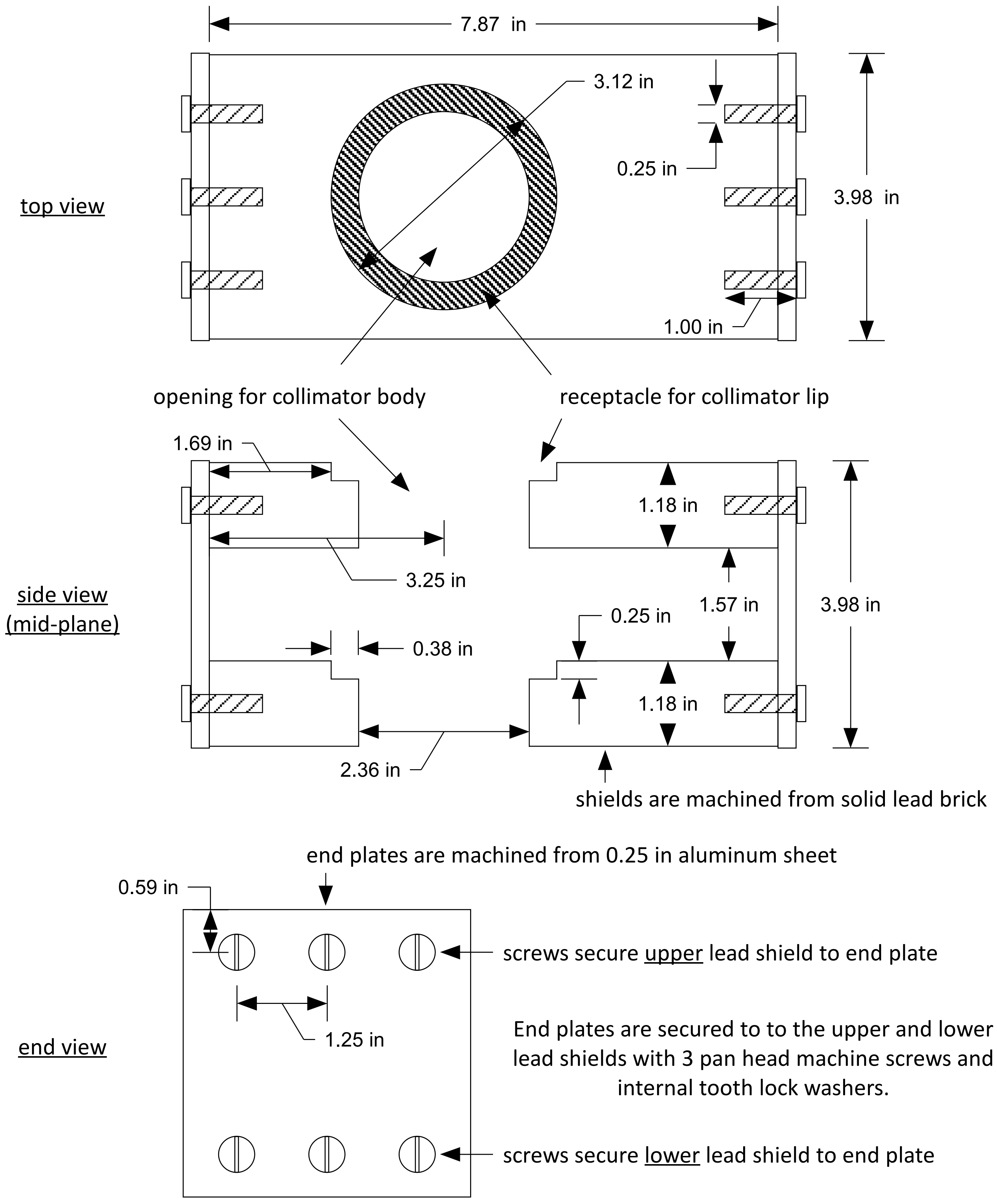

Circular Collimators
(drawn at 1:2 scale)

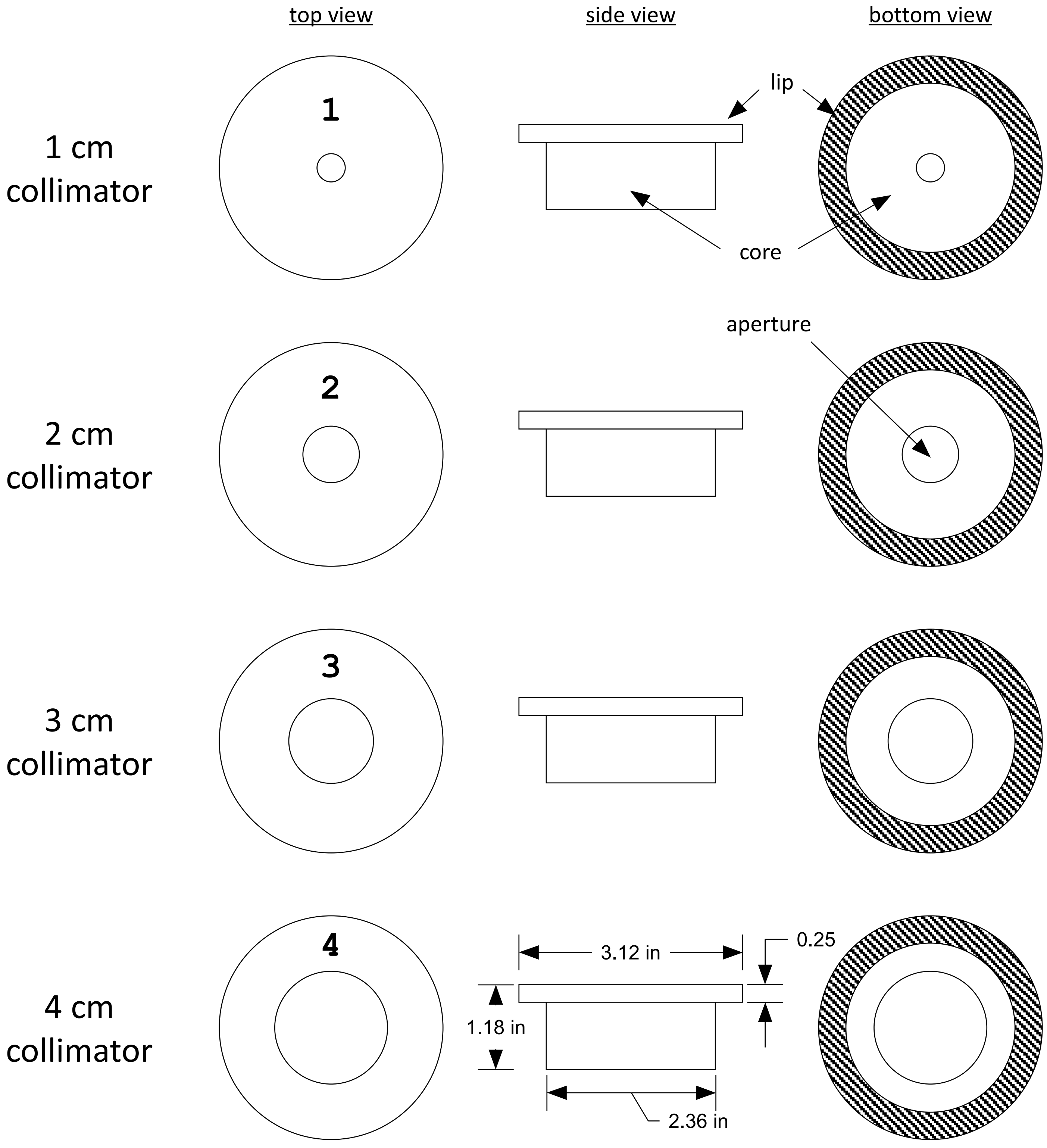

collimators are machined from solid lead bar

base plate for restrainer
(drawn at 1:2 scale)

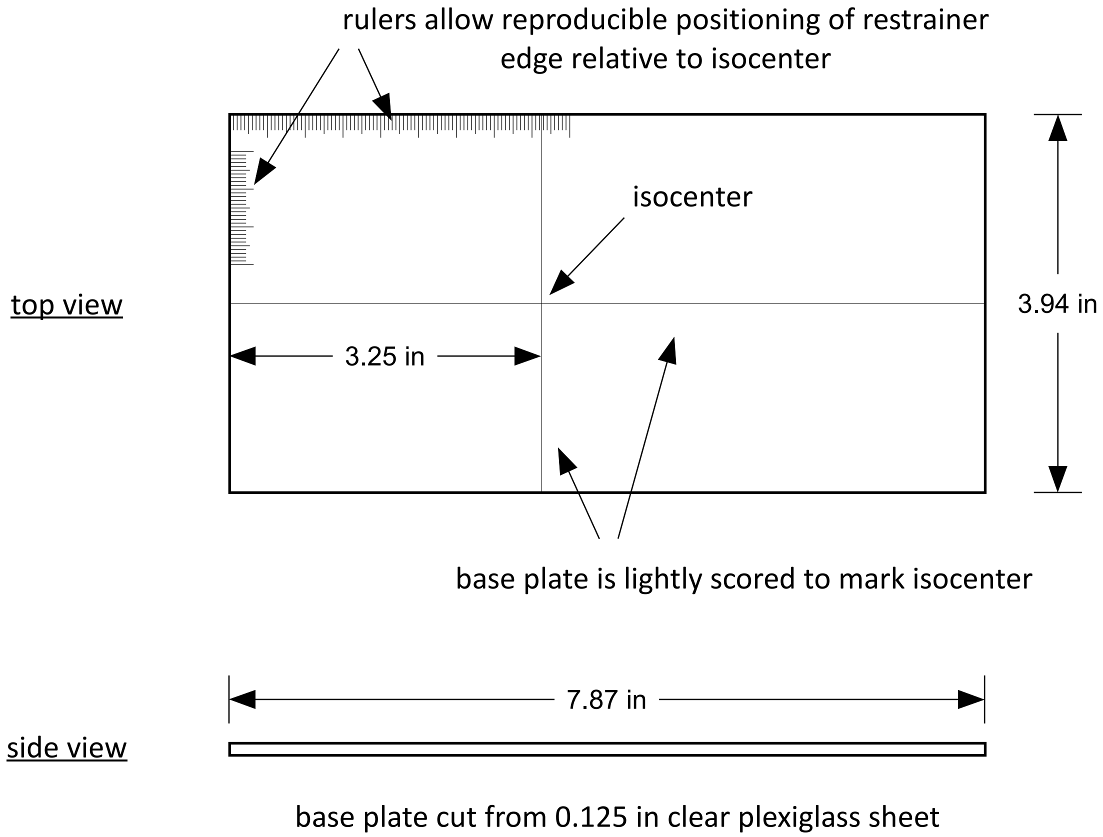

Lead shield with circular collimator assembly and use instructions

(2 cm collimator is shown)

side view

top view

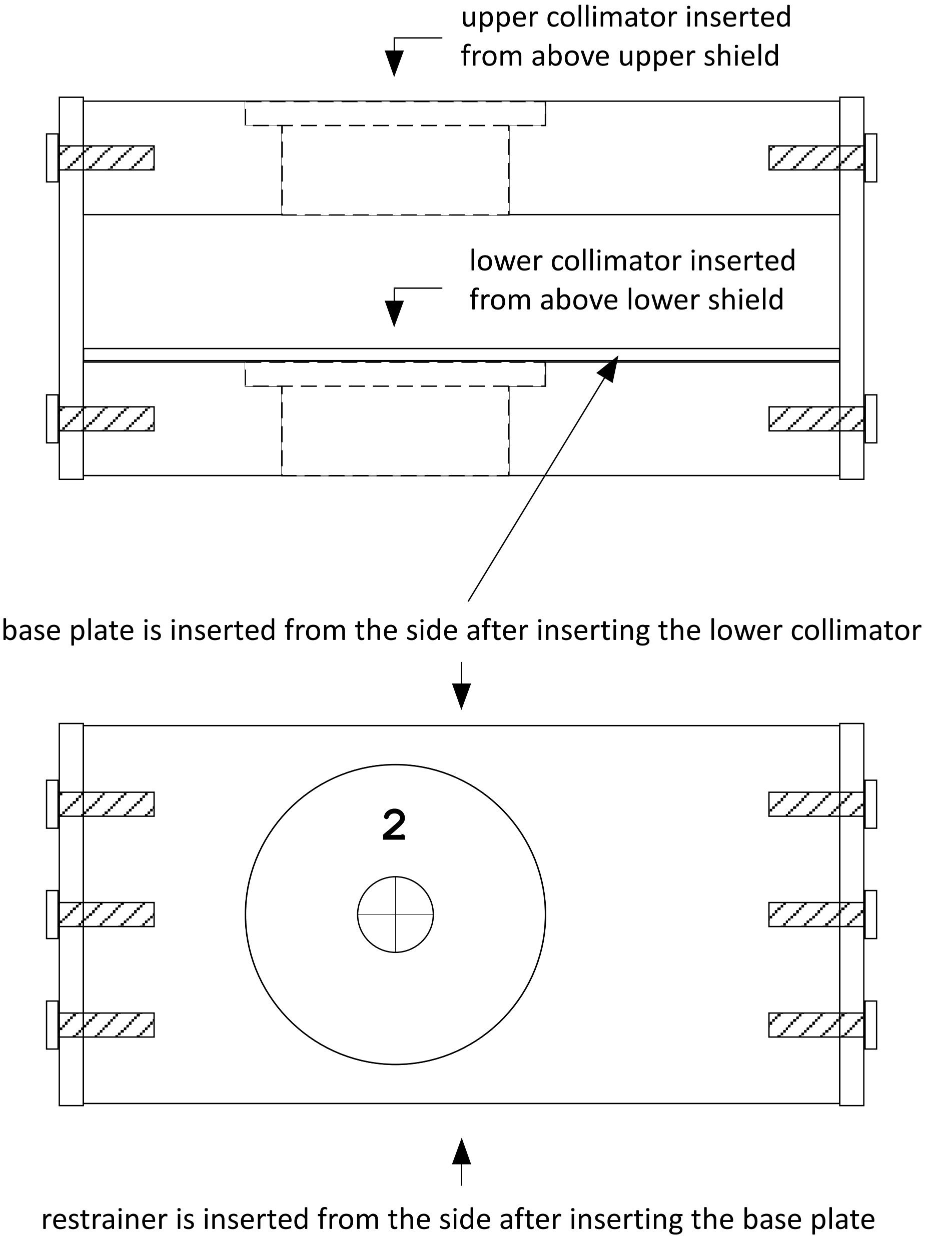

cradle for lead shield/collimator
(drawn at 1:2 scale)

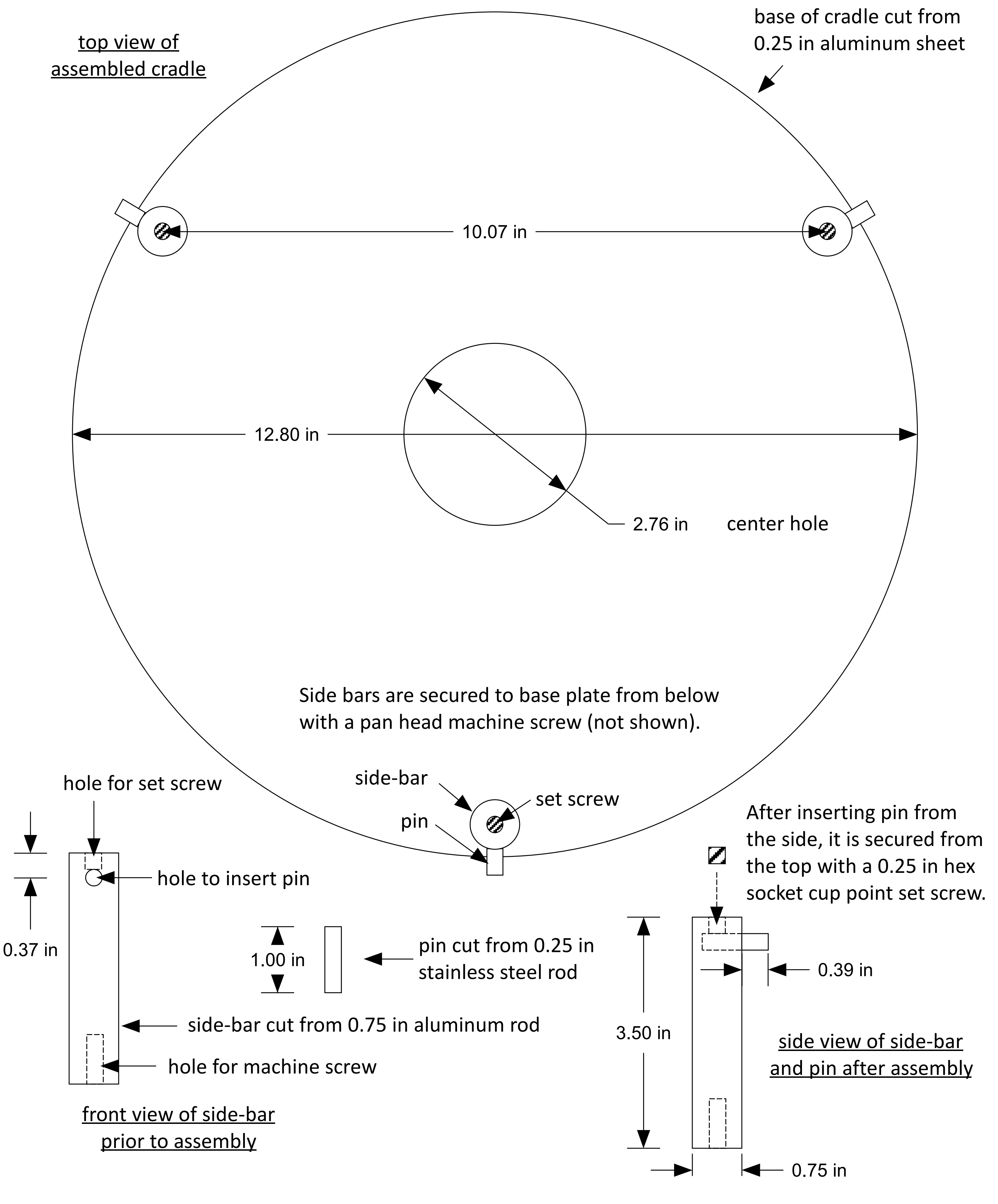

schematic of collimated radiation field (drawn at 1:4 scale)

Note: the outer enclosures of the radiation chamber are not shown for clarity

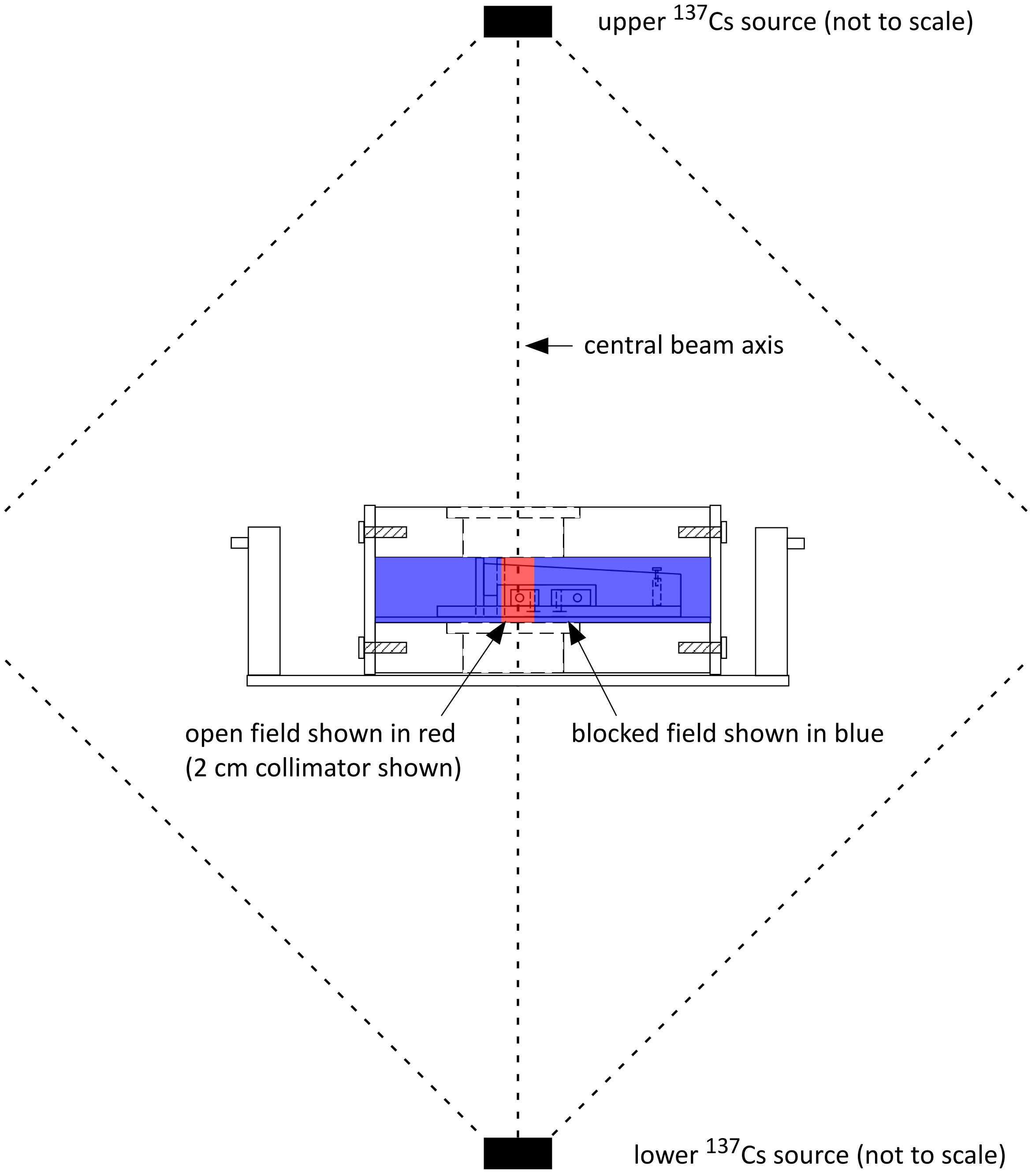

Lead shield
(drawn at 1:2 scale)

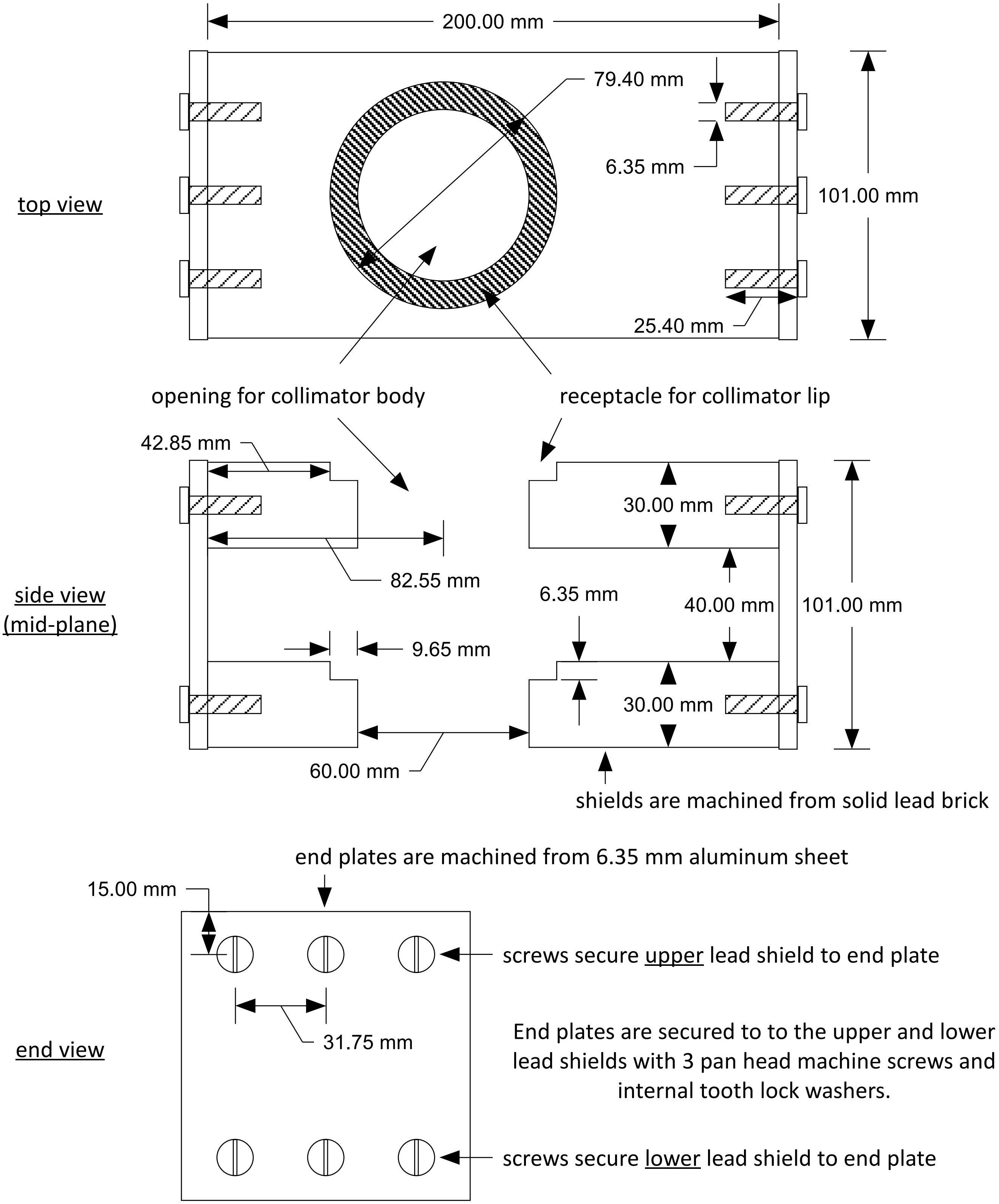

Circular Collimators
(drawn at 1:2 scale)

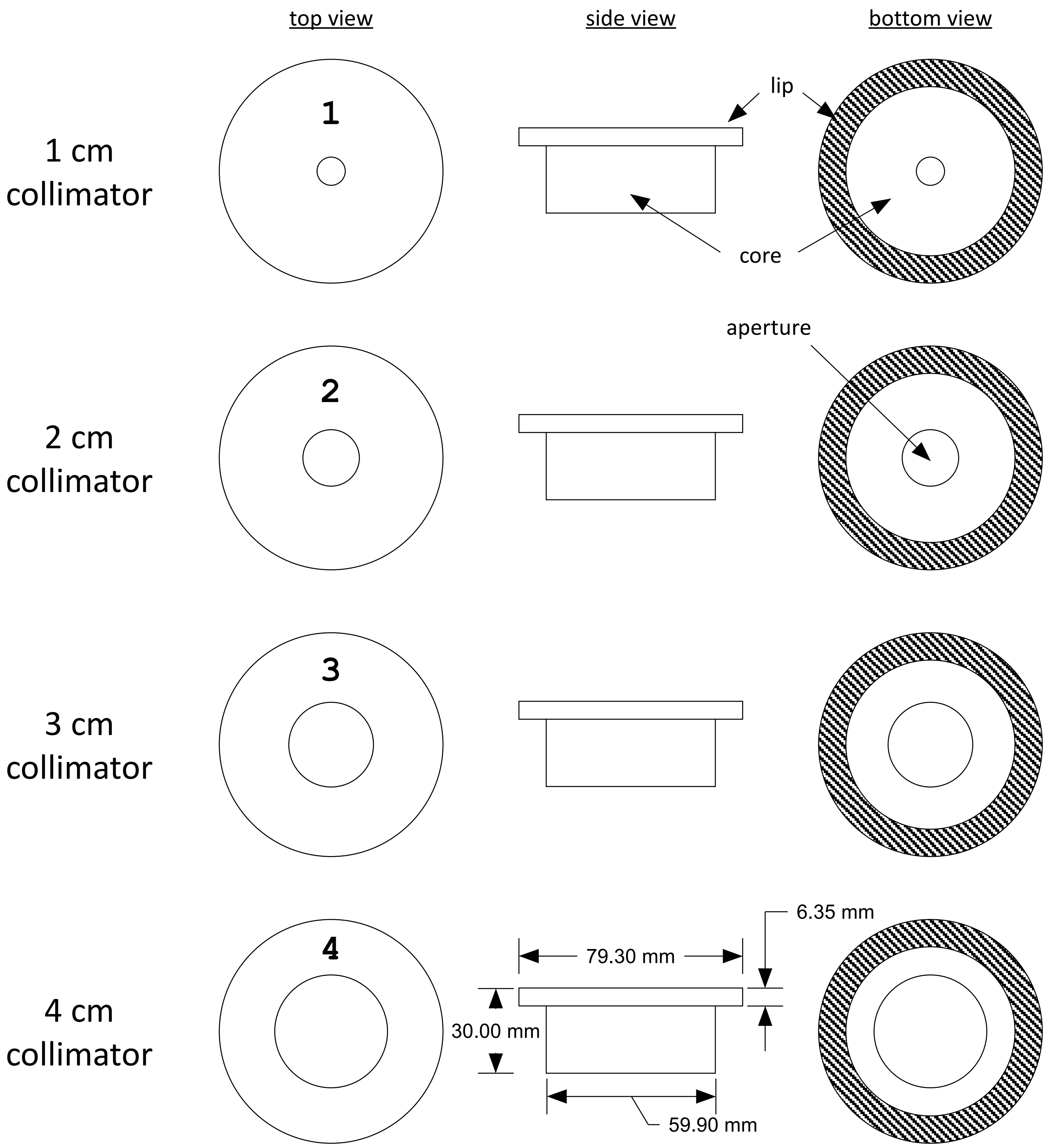

collimators are machined from solid lead bar

base plate for restrainer
(drawn at 1:2 scale)

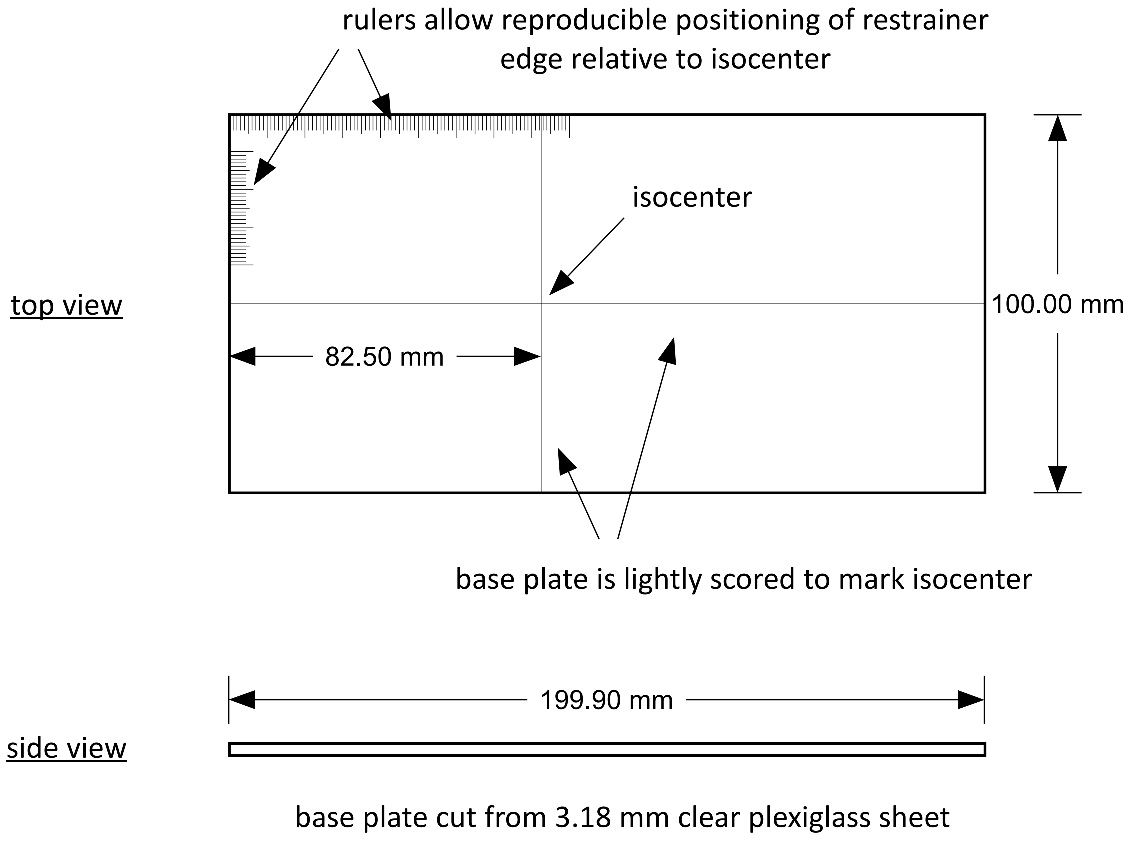

Lead shield with circular collimator assembly and use instructions

(2 cm collimator is shown)

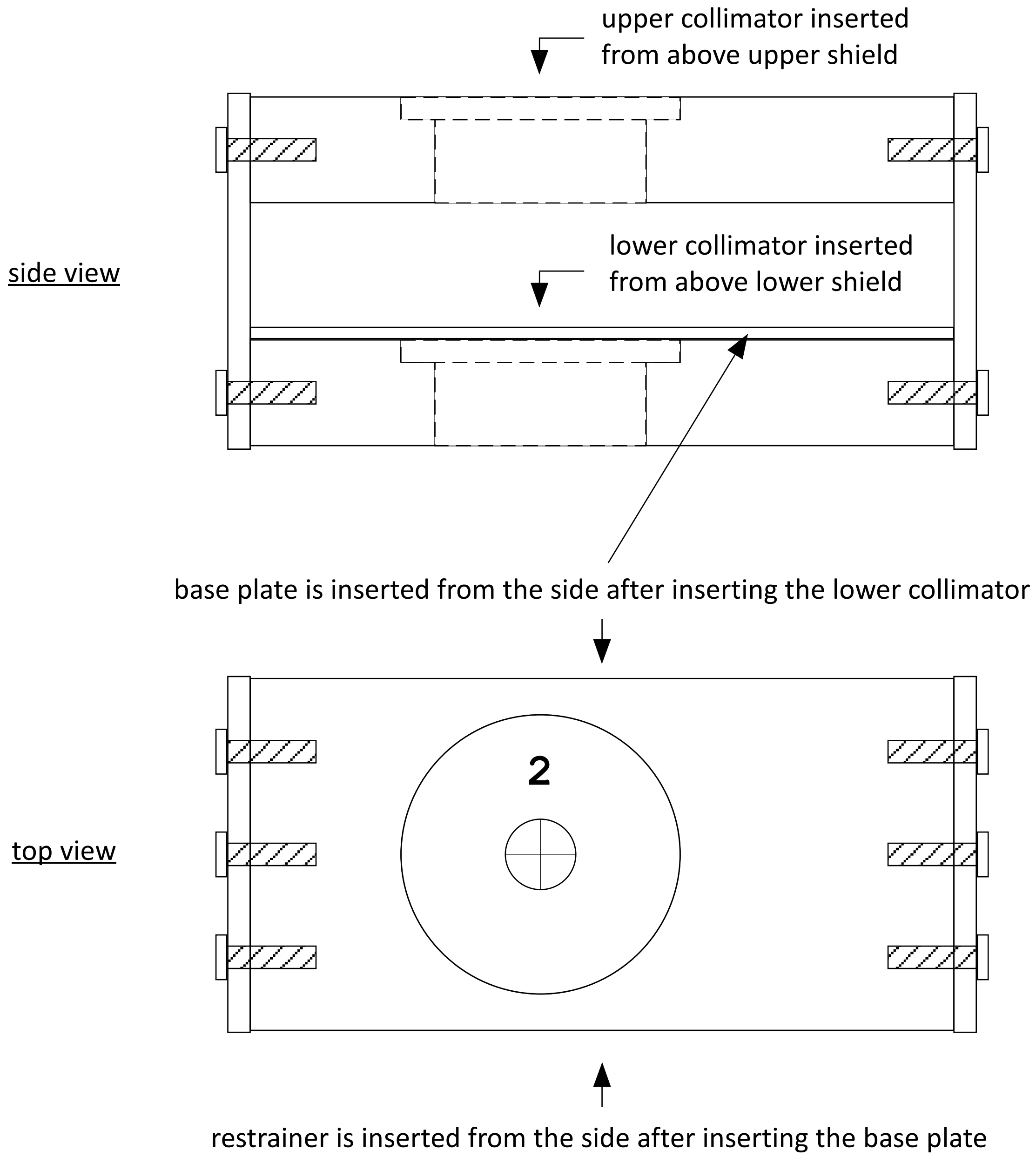

cradle for lead shield/collimator
(drawn at 1:2 scale)

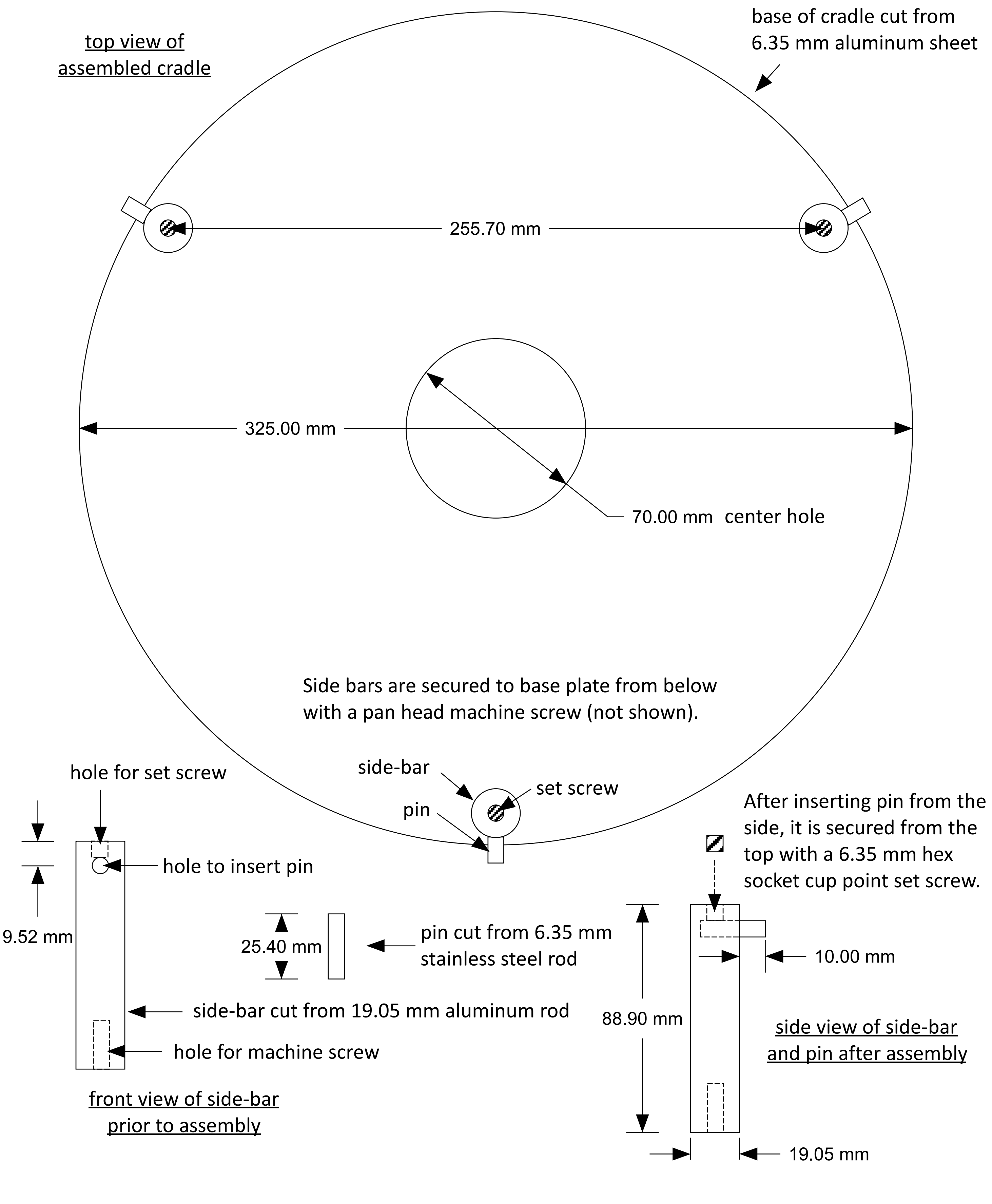

schematic of collimated radiation field (drawn at 1:4 scale)

Note: the outer enclosures of the radiation chamber are not shown for clarity

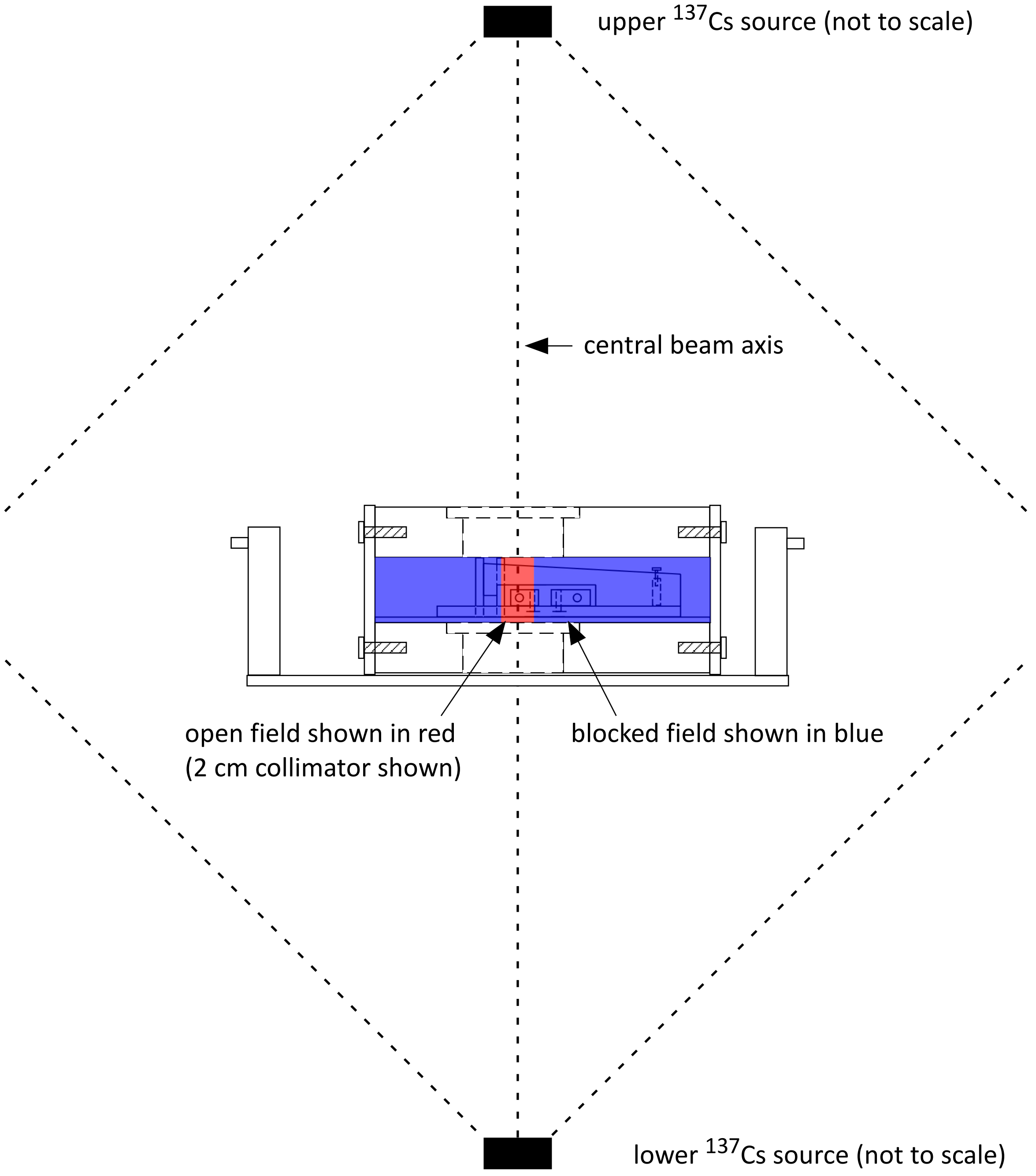

Supplement: Supplementary file 1 — Supplementary Information [file 43856_2023_336_MOESM1_ESM.pdf]
