# Supplementary material for: Ablative radiotherapy improves survival but does not cure autochthonous mouse models of prostate and colorectal cancer
Source: Commun Med (Lond). 2023 Aug 9;3:108. doi: 10.1038/s43856-023-00336-3 (PMC10412558; doi:10.1038/s43856-023-00336-3)
Supplement: Supplementary file 5 — Description of Additional Supplementary Files [file 43856_2023_336_MOESM5_ESM.pdf]

Description of Additional Supplementary Files

File Name: Supplementary Data 1

Description: Source data

File Name: Supplementary Video 1

Description: This 24 second movie shows an approximately 5-minute fluoroscopic image time-lapse (12X speed) of a mouse in the restrainer imaged by μCT. IV contrast was used to aid in visualization of kidneys and bladder
