# Supplementary material for: Ablative radiotherapy improves survival but does not cure autochthonous mouse models of prostate and colorectal cancer
Source: Commun Med (Lond). 2023 Aug 9;3:108. doi: 10.1038/s43856-023-00336-3 (PMC10412558; doi:10.1038/s43856-023-00336-3)
Supplement: Supplementary file 6 — Reporting Summary [file 43856_2023_336_MOESM6_ESM.pdf]

nature portfolio

Corresponding author(s): Daniel Schmidt and Matthew Vander Heiden

Last updated by author(s): Jun 14, 2023

Reporting Summary

Nature Portfolio wishes to improve the reproducibility of the work that we publish. This form provides structure for consistency and transparency in reporting. For further information on Nature Portfolio policies, see our Editorial Policies and the Editorial Policy Checklist.

Statistics

For all statistical analyses, confirm that the following items are present in the figure legend, table legend, main text, or Methods section.

n/a	Confirmed	
☐	☒	The exact sample size (n) for each experimental group/condition, given as a discrete number and unit of measurement
☐	☒	A statement on whether measurements were taken from distinct samples or whether the same sample was measured repeatedly
☐	☒	The statistical test(s) used AND whether they are one- or two-sided<br>*Only common tests should be described solely by name; describe more complex techniques in the Methods section.*
☒	☐	A description of all covariates tested
☐	☒	A description of any assumptions or corrections, such as tests of normality and adjustment for multiple comparisons
☐	☒	A full description of the statistical parameters including central tendency (e.g. means) or other basic estimates (e.g. regression coefficient) AND variation (e.g. standard deviation) or associated estimates of uncertainty (e.g. confidence intervals)
☒	☐	For null hypothesis testing, the test statistic (e.g. F, t, r) with confidence intervals, effect sizes, degrees of freedom and P value noted<br>*Give P values as exact values whenever suitable.*
☒	☐	For Bayesian analysis, information on the choice of priors and Markov chain Monte Carlo settings
☒	☐	For hierarchical and complex designs, identification of the appropriate level for tests and full reporting of outcomes
☒	☐	Estimates of effect sizes (e.g. Cohen's d, Pearson's r), indicating how they were calculated

Our web collection on statistics for biologists contains articles on many of the points above.

Software and code

Policy information about availability of computer code

Data collection	No custom software or computer code was used for data collection.
Data analysis	All software used for data analysis is commercially available or on an open source server. The developer/vendor and version has been identified in the manuscript. No custom computer code was generated for data analysis.

For manuscripts utilizing custom algorithms or software that are central to the research but not yet described in published literature, software must be made available to editors and reviewers. We strongly encourage code deposition in a community repository (e.g. GitHub). See the Nature Portfolio guidelines for submitting code & software for further information.

Data

Policy information about availability of data

All manuscripts must include a data availability statement. This statement should provide the following information, where applicable:

- Accession codes, unique identifiers, or web links for publicly available datasets
- A description of any restrictions on data availability
- For clinical datasets or third party data, please ensure that the statement adheres to our policy

All data are available in the main text or the supplementary materials.

Human research participants

Policy information about studies involving human research participants and Sex and Gender in Research.

Reporting on sex and gender	N/A
Population characteristics	N/A
Recruitment	N/A
Ethics oversight	N/A

Note that full information on the approval of the study protocol must also be provided in the manuscript.

Field-specific reporting

Please select the one below that is the best fit for your research. If you are not sure, read the appropriate sections before making your selection.

☒ Life sciences ☐ Behavioural & social sciences ☐ Ecological, evolutionary & environmental sciences

For a reference copy of the document with all sections, see nature.com/documents/nr-reporting-summary-flat.pdf

Life sciences study design

All studies must disclose on these points even when the disclosure is negative.

Sample size	No formal a priori sample size calculations were performed for this study. Sample sizes were chosen based on biological variation observed in pilot studies and were designed to demonstrate biologically relevant differences between groups. The sample sizes in this study are in line with similar studies reported in scientific literature.
Data exclusions	No data were excluded from the analyses.
Replication	All replicate data is as reported in the manuscript. No replicate data has been excluded.
Randomization	Assignment of animals to treatment groups was random.
Blinding	Whenever possible, and where appropriate, data collection and analysis was performed in a blinded fashion.

Reporting for specific materials, systems and methods

We require information from authors about some types of materials, experimental systems and methods used in many studies. Here, indicate whether each material, system or method listed is relevant to your study. If you are not sure if a list item applies to your research, read the appropriate section before selecting a response.

Materials & experimental systems

n/a	Involved in the study	
☐	☒	Antibodies
☐	☒	Eukaryotic cell lines
☒	☐	Palaeontology and archaeology
☐	☒	Animals and other organisms
☒	☐	Clinical data
☒	☐	Dual use research of concern

Methods

n/a	Involved in the study	
☒	☐	ChIP-seq
☐	☒	Flow cytometry
☒	☐	MRI-based neuroimaging

Antibodies

Antibodies used	See methods section.
Validation	All antibodies used in the study have been reported by the manufacturer to work on mouse.

Eukaryotic cell lines

Policy information about cell lines and Sex and Gender in Research

Cell line source(s)	source of established prostate cancer cell lines are described in the methods section
Authentication	STR testing
Mycoplasma contamination	cell lines were routinely tested for mycoplasma
Commonly misidentified lines (See ICLAC register)	N/A

Animals and other research organisms

Policy information about studies involving animals; ARRIVE guidelines recommended for reporting animal research, and Sex and Gender in Research

Laboratory animals	Species, strain, and age are reported in the methods section and/or figure legends.
Wild animals	N/A
Reporting on sex	For prostate cancer model studies only male mice were used. For all other studies both male and female mice were used.
Field-collected samples	N/A
Ethics oversight	Animal studies were approved by the MIT Institutional Animal Care and Use Committee

Note that full information on the approval of the study protocol must also be provided in the manuscript.

Flow Cytometry

Plots

Confirm that:

☒ The axis labels state the marker and fluorochrome used (e.g. CD4-FITC).

☒ The axis scales are clearly visible. Include numbers along axes only for bottom left plot of group (a 'group' is an analysis of identical markers).

☒ All plots are contour plots with outliers or pseudocolor plots.

☒ A numerical value for number of cells or percentage (with statistics) is provided.

Methodology

Sample preparation	Blood, spleens, pelvic LNs, and axillary LNs were harvested 2 days or 8 weeks after the last dose of radiation. All tissue samples were weighed and kept in RPMI media (ATCC) on ice during collection. Blood was collected from the abdominal vena cava or aorta into K2-EDTA tubes (Grenier-Bio) and kept on ice. Spleens and LNs were mechanically digested through 70 um nylon cell strainers to prepare single-cell suspensions for staining. Red blood cells in spleen and blood samples were lysed in ACK Lysis Buffer (Gibco). All samples were resuspended in ice-cold PBS for viability staining and ice-cold PBS containing 1% (w/v) BSA and 2 mM EDTA (FACS buffer) for extracellular labeling. Intracellular staining and fixation was performed using the FoxP3 Transcription Factor Buffer Set (eBioscience).
Instrument	Cells were analyzed using BD FACS LSR Fortessa or BD FACS Symphony A3 flow cytometers.
Software	BD FACSDiva (BD Biosciences) was used for the collection of flow cytometry data. FlowJo was used for analysis. The collected data were plotted with statistical analysis by GraphPad Prism.
Cell population abundance	No sorting was used in this study.
Gating strategy	Representative gating is provided in Supplementary Data Figure 5D

☒ Tick this box to confirm that a figure exemplifying the gating strategy is provided in the Supplementary Information.
